# Microbial biosynthesis of rare cannabinoids

**DOI:** 10.1093/jimb/kuaf013

**Published:** 2025-05-13

**Authors:** Chunsheng Yan, Ikechukwu C Okorafor, Colin W Johnson, Kendall N Houk, Neil K Garg, Yi Tang

**Affiliations:** Department of Chemical and Biomolecular Engineering, University of California, Los Angeles, CA 90095, USA; Department of Chemical and Biomolecular Engineering, University of California, Los Angeles, CA 90095, USA; California NanoSystems Institute, University of California, Los Angeles, CA 90095, USA; Department of Chemistry and Biochemistry, University of California, Los Angeles, CA 90095, USA; Department of Chemistry and Biochemistry, University of California, Los Angeles, CA 90095, USA; Department of Chemistry and Biochemistry, University of California, Los Angeles, CA 90095, USA; Department of Chemical and Biomolecular Engineering, University of California, Los Angeles, CA 90095, USA; Department of Chemistry and Biochemistry, University of California, Los Angeles, CA 90095, USA

**Keywords:** Polyketide, Natural product, Cannabinoids, Prenyltransferase, Yeast

## Abstract

∆^9^–tetrahydrocannabinol (∆^9^–THC) and cannabidiol are the most abundant natural cannabinoids isolated from the different cultivars of the *Cannabis* plant. Other natural ∆^9^–THC analogs, especially those with different alkyl chain substitutions, display different and potent bioactivity. However, these rare cannabinoids are typically isolated in minuscule amounts and are difficult to synthesize. Targeted microbial biosynthesis can therefore be an attractive route to access such molecules. Here, we report the development of a *Saccharomyces cerevisiae* host to biosynthesize 2 rare cannabinoids from simple sugars. The yeast host is engineered to accumulate excess geranyl pyrophosphate, to overexpress a fungal pathway to 2,4-dihydroxy-6-alkyl-benzoic acids, as well as the downstream UbiA-prenyltransferase and ∆^9^–tetrahydrocannabinolic acid synthase. Two rare cannabinoid acids, the C1-substituted ∆^9^–tetrahydrocannabiorcolic acid (∼16 mg/L) and the C7-substituted ∆^9^–tetrahydrocannabiphorolic acid (∼5 mg/L) were obtained from this host; the latter was thermally decarboxylated to give ∆^9^–tetrahydrocannabiphorol. Given the diversity of fungal biosynthetic gene clusters that can produce resorcylic acids, this microbial platform offers the potential to produce other rare and new-to-nature cannabinoids.

**One Sentence Summary:**  *Saccharomyces cerevisiae* as a host to produce rare cannabinoids.

## Graphical Abstract

**Graphical Abstract:**
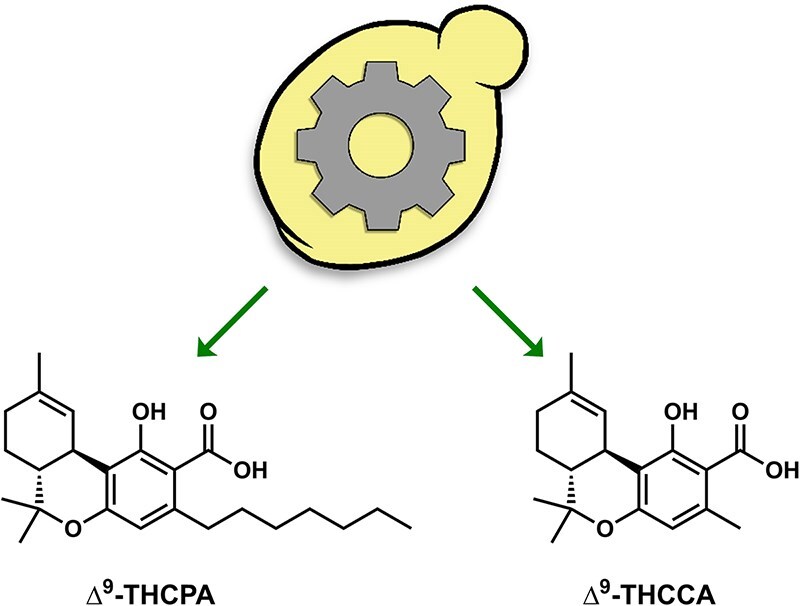

## Introduction

**Fig. 1.**
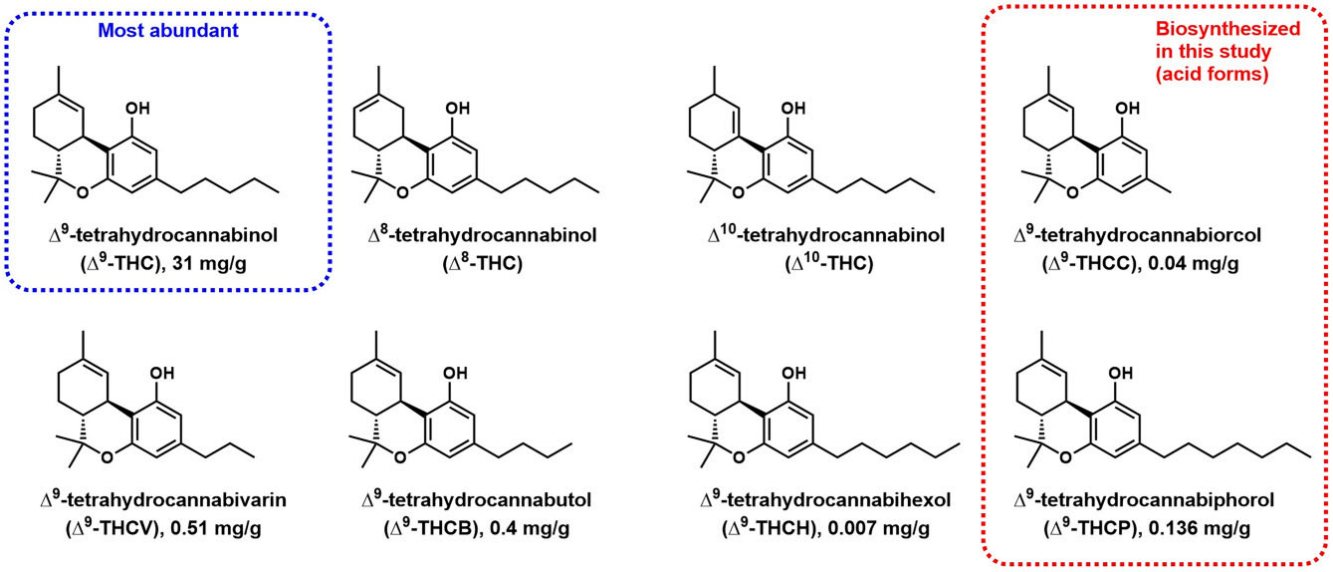
∆^9^ –THC and other natural ∆^9^–THC analogs isolated from natural *Cannabis* plant. The reported titers per g of plant biomass are listed. The acid forms of 2 rare analogs, ∆^9^–THCCA and ∆^9^–THCPA, are biosynthesized from yeast in this work.

Cannabinoids are a large class of bioactive natural products that interact with the cannabinoid receptors of the human endocannabinoid system (Pertwee, 2006). The most abundant and well-known natural cannabinoids are ∆^9^–tetrahydrocannabinol (∆^9^–THC) and cannabidiol (CBD), isolated from different cultivars of the *Cannabis* plant. Cannabinoid-based medicines (CBMs) have shown promise as pharmacological agents, acting as antidepressants, analgesics, anticonvulsants, and antiemetics (Devinsky et al., 2014). Two CBMs, cesamet (nabilone), an antiemetic for cancer chemotherapy (Dalzell et al., 1986), and dronabinol, an antiemetic that also treats appetite loss, are currently on the market (Patar et al., 2023). Rare cannabinoids with different alkyl chain substitutions instead of the 5-carbon (C5) chain in ∆^9^–THC and CBD have been isolated from plant sources (Adama et al., 1942; Gill et al., 1970; Hanus & Krejci, 1975; Srebnik et al., 1984; Citti et al., 2019; Linciano et al., 2020a, 2020b), some of which display stronger affinity toward human CB_1_ receptors than ∆^9^–THC (Fig. 1). For example, ∆^9^–tetrahydrocannabutol (∆^9^–THCB) with a butyl (C4) chain and ∆^9^-tetrahydrocannabiphorol (∆^9^–THCP) with a heptyl (C7) chain display *K*_i_ values of 15 nM (Linciano et al., 2020a) and 1.2 nM (Citti et al., 2019), respectively, against human CB1 receptor *in vitro*, which correspond to ∼5-fold and 30-fold stronger inhibition compared to that of ∆^9^–THC (*K*_i_ = 40 nM) (Citti et al., 2019). While these natural, rare cannabinoids are attractive lead molecules for CBMs, the low abundance of these molecules poses significant challenges to scalable isolation (ElSohly & Gul, 2014). From dried and decarboxylated raw plant materials, ∼ 30 mg/g of the most abundant cannabinoid ∆^9^–THC can be isolated (Backman, 2023). In contrast, the compositions of other ∆^9^–THC analogs are all below 1 mg/g (Fig. 1) (Ross et al., 2005; Citii et al., 2019; Linciano et al., 2020a, 2020b). Synthetic methods for accessing the cannabinoid molecules have been developed, but remain difficult to implement at scale due to the complexities involved in the multistep reactions (Bloemendal et al., 2020). As a result, synthetic biology approaches utilizing microbial hosts are attractive options for obtaining rare and new-to-nature cannabinoid molecules.

**Fig. 2.**
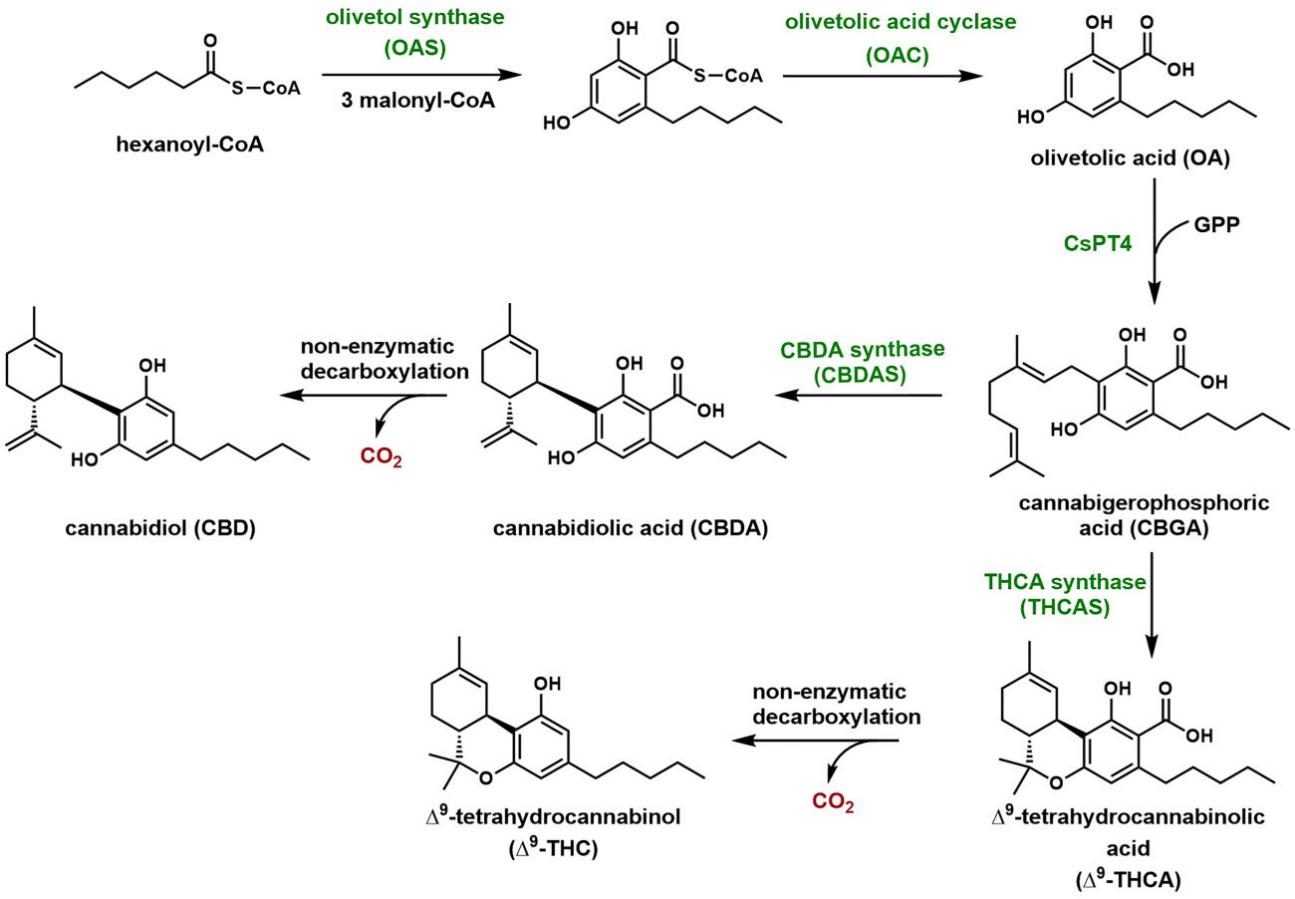
The plant biosynthetic pathway to CBD and ∆^9^–THC. The pathway has been introduced into microbes for partial and complete pathway expression.

Various strategies toward engineering microbes to produce cannabinoids have been reported since the identification of *Cannabis sativa L*. plant olivetolic acid synthase (OLS) and olivetolic acid cyclase (OAC) (Gülck & Møller, 2020), which collectively synthesize olivetolic acid (OA), the C5 substituted resorcylic acid intermediate (Fig. 2). Gonzalez and coworkers achieved 80 mg/L production of OA upon overexpression of OLS and OAC in *E. coli* (Tan et al., 2018). In a cell-free synthetic biology approach, Bowie and coworkers engineered a *Streptomyces* prenyltransferase, NphB, to geranylate OA regioselectively to produce cannabigerolic acid (CBGA) (Valliere et al., 2020). Keasling and coworkers identified a *Cannabis sativa* prenyltransferase, CsPT4, that natively performs *C*-geranylation of OA to CBGA. Further co-expression with tetrahydrocannabinolic acid cyclase (THCAS) (Sirikantaramas et al., 2004) enabled reconstitution of the complete cannabinoid pathway in *S. cerevisiae* that produced 8 mg/L of ∆^9^–tetrahydrocannabinolic acid (∆^9^–THCA), with 1.2 mg/L ∆^9^–tetrahydrocannabivarinic acid produced as a side product (Fig. S1) (Luo et al., 2019). This yeast system was further improved to increase precursor supply and CsPT4 function to produce CBGA at 510 mg/L (Zhang et al., 2023). All these engineering efforts are based on the plant pathway and aimed to improve the production of the two major precursors, hexanoyl-CoA and geranyl pyrophosphate (GPP).

**Fig. 3.**
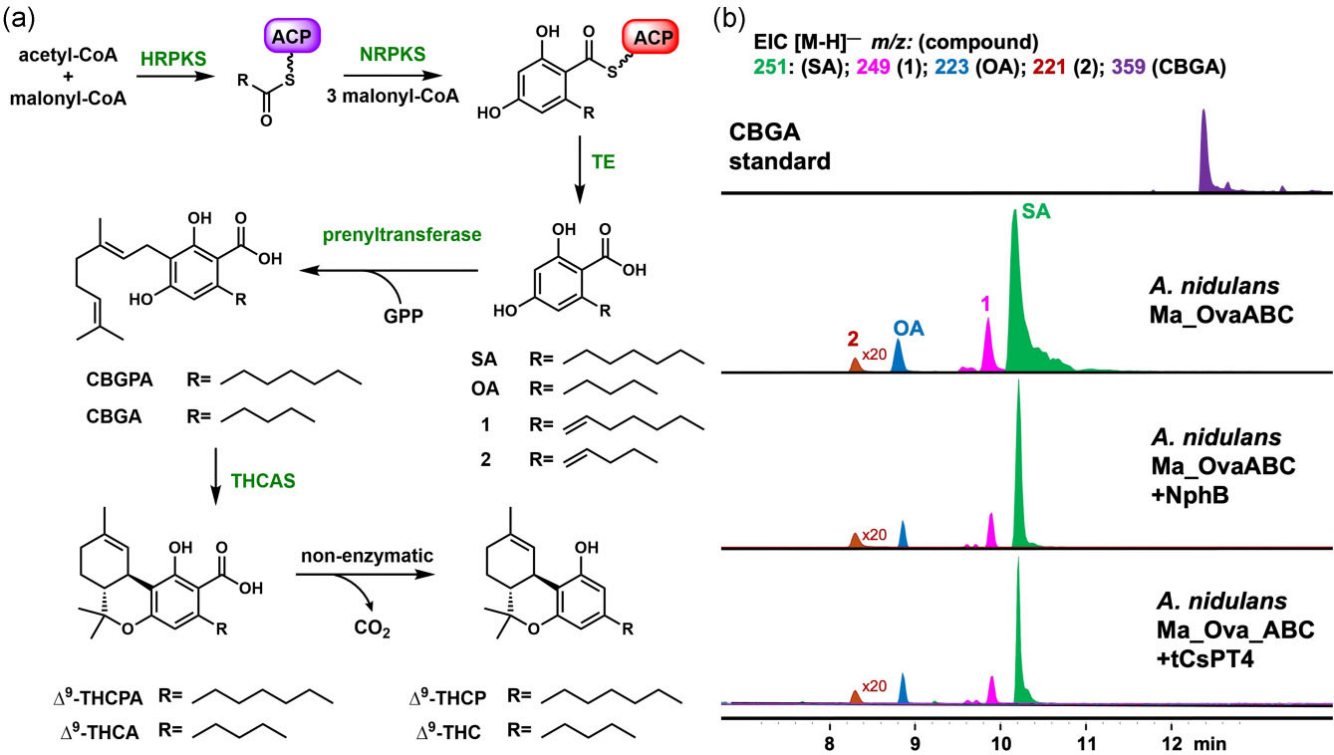
Proposed pathway to access rare cannabinoids such as ∆^9^–THCPA with the C7 side chain. (a) Proposed extension of the biosynthetic pathway involving Ma_OvaABC to produce ∆^9^–THC and analogs; (b) Metabolic analysis from the heterologous expression of Ma_OvaABC and tCsPT4, NphB in *A. nidulans* shows the prenyltransferases do not function in the fungal host.

Our laboratory previously reported a fungal biosynthetic pathway that can synthesize OA and other alkyl-substituted 2,4-dihydroxybenzoic acids. The 3-enzyme pathway, derived from *Metarhizium anisopliae*, consists of a highly reducing polyketide synthase (HRPKS, Ma_OvaA) that generates the alkyl chain starter unit, a nonreducing polyketide synthase (NRPKS, Ma_OvaB) that elongates and cyclizes the starter unit into the 2,4-dihydroxy-6-alkyl benzoyl-thioester, and a thioesterase (Ma_OvaC) that releases the product (Fig. 3a) (Okorafor et al., 2021). When heterologously expressed in the *Aspergillus nidulans* A1145 ΔSTΔEM host (Yee & Tang, 2022), the pathway produced the C7 sphaerophorolcarboxylic acid (SA) with a titer of 1.4 g/L, along with OA at 80 mg/L, and unsaturated analogs of SA and OA, which are **1** (∼140 mg/L) and **2** (0.4 mg/L), respectively (Fig. 3b) (Okorafor et al., 2021). This fungal biosynthetic pathway therefore provides direct access to SA at high titer and can be a starting point to access the rare but highly potent C7 ∆^9^–THCP (Fig. 1) (Citii et al. 2019). In this report, we demonstrate the reconstitution of this pathway in an engineered yeast host, which enabled the *in vivo* production of ∆^9^–tetrahydrocannabiphorolic acid (∆^9^–THCPA) that can be subsequently decarboxylated to ∆^9^–THCP, along with the unexpected production of C1-substituted ∆^9^–tetrahydrocannabiorcolic acid (∆^9^–THCCA).

## Results and Discussion

### Attempted Pathway Engineering in A. nidulans

Upon expressing the biosynthetic gene cluster containing the Ma_OvaABC genes in *A. nidulans*, multiple OA analogs were produced (Fig. 3b). Extension of this pathway with co-expression of prenyltransferase and the cyclase THCAS should therefore lead to CBGA and THCA analogs. To investigate if prenylation reaction can be reconstituted, a thermal-stabilized version of the bacterial prenyltransferase NphB, engineered for efficient prenylation of OA to CBGA (Valliere et al., 2020), was expressed in *A. nidulans* with Ma_OvaABC (Fig. 3b). Upon metabolite analysis, NphB did not prenylate OA or other alkyl-substituted 2,4-dihydroxybenzoic acids. Previous reports suggested the soluble ABBA-type prenyltransferase NphB is highly active *in vitr o* (Valliere et al., 2020), but activity is significantly reduced when expressed in *Komagataella phaffii* (Zirpel et al., 2017). We hypothesized that the soluble NphB may be localized in the cytoplasm of *A. nidulans* and therefore has limited access to the key substrate, geranyl pyrophosphate, produced by the mevalonate pathway, which involves ER-localized enzymes and other enzymes localized in different cellular regions (Orban & Rühl, 2022). To investigate the subcellular localization of NphB in *A. nidulans*, we performed intracellular localization analysis by fusing NphB to a C-terminal green fluorescent protein (GFP). A flexible amino acid linker (GGSGG) sequence was inserted between NphB and GFP (Chen et al., 2013). Microscopic images of the tagged NphB enzyme in *A. nidulans* showed that NphB was not localized in any punctuate organelles but rather was distributed throughout the fungal body, indicating that the enzyme is located in the cytoplasm, which potentially limits access to GPP substrate and hence lack of prenylation activity in *A. nidulans* (Fig. S2).

CsPT4 is a *Cannabis* plant enzyme that belongs to the UbiA-family of membrane-bound prenyltransferases. This enzyme was shown to catalyze geranylation of OA in yeast to afford CBGA (Luo et al., 2019). To assess its activity in *A. nidulans*, we coexpressed a truncated CsPT4 without the plastid targeting sequence (tCsPT4) with Ma_OvaABC in *A. nidulans* (Luo et al., 2019). However, no prenylated products were observed (Fig. 3b). *Aspergillus nidulans* was previously used for monoterpene and diterpene production, indicating there is sufficient GPP in the host (Bromann et al., 2016). Furthermore, using a monoterpene pathway as a test, we verified that GPP supply is available in the fungal host (data not shown). *Aspergillus nidulans* has not been commonly used for the heterologous expression of plant-derived enzymes, which may be due to different intracellular environment or localization compared to plant hosts. These factors may affect the stability of heterologous enzymes such as tCsPT4. Collectively, we concluded that further attempts to extend the cannabinoid pathway in *A. nidulans* are not warranted and decided to move the pathway into the yeast *S. cerevisiae*.

### Pathway Engineering in Yeast

**Fig. 4:**
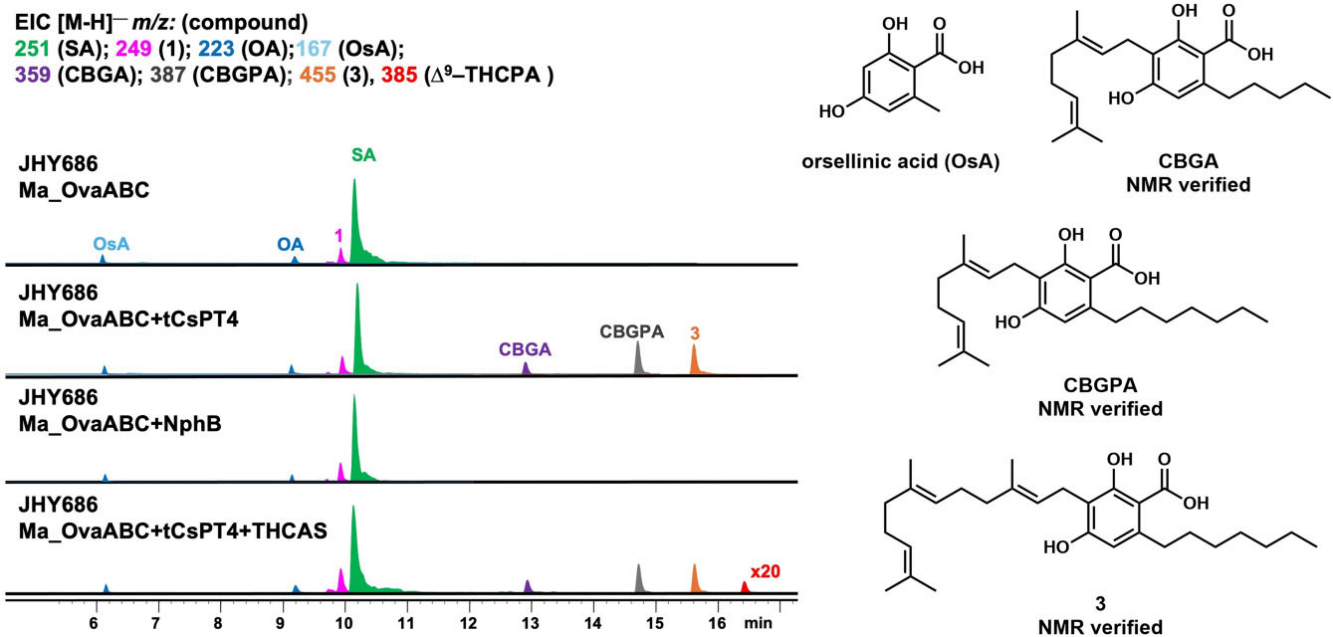
Heterologous expression Ma_OvaABC, tCsPT4, and THCAS in *S. cerevisiae* JHY686 strain. Expression of Ma_OvaABC in JHY686 resulted in production of SA. Co-expression of tCsPT4 with Ma*_*OvaABC led to the biosynthesis of CBGPA along with the farnesyl pyrophosphate (FPP)-derived shunt product **3**. The presence of orsellinic acid (OsA) was proposed based on the molecular weight (MWT) and UV absorbance. The structures of the isolatable compounds are verified by NMR. In contrast, co-expression of NphB with Ma_OvaABC did not lead to prenylated products. Co-expression of tCsPT4 and THCAS with Ma_OvaABC led to trace amounts of a compound with MWT of 386, same as ∆^9^–THCPA.

The Ma_OvaABC pathway was first expressed in the *S. cerevisiae* strain JHY686, a strain derived from the integration of the *A. nidulans* phosphopantetheinyl (pPant) transferase into the chromosome of the JHY651 strain (Table S3) (Yee et al., 2019). Each enzyme was expressed from anautoinducible ADH promoter on a 2µm plasmid (Harvey et al., 2018). The resulting yeast strain, after 4 days of culturing in YPD media, was extracted and analyzed for metabolite production (Fig. 4). Multiple 2,4-dihydroxybenzoic acid compounds, including OA, SA, **1**, and a compound with molecular weight (MWT) identical to orsellinic acid (OsA) were detected. The titer of the most abundant compound, SA, was determined to be ∼500 mg/L in this unoptimized strain. Although the titer is lower than that in *A. nidulans* (1.4 g/L), the robust biosynthesis of SA in yeast is an encouraging first step in downstream pathway construction.

Co-expression of NphB with Ma_OvaABC in JHY686 again did not produce any prenylated products (Fig. 3). However, co-expression of tCsPT4 with Ma_OvaABC led to the production of CBGA (as compared to a standard) and 2 new compounds with MWT of 388 (∼5 mg/L) and 456 (∼8 mg/L). To elucidate the structures of the two compounds, large-scale yeast culturing (5 × 800 mL) was performed, followed by purification. Full nuclear magnetic resonance (NMR) characterization revealed that the compound with MWT of 388 was indeed geranylated SA product cannabigerophorolic acid (CBGPA) (Table S4, Figs S7–S12), while the compound with MWT of 456 was the farnesylated SA (**3**) derived from transfer of farnesyl group from farnesyl diphosphate (FPP) to SA (Fig. 3, Table S7, Figs S25–S30). Successful prenylation of OA and SA in the yeast host, albeit incomplete, led us to coexpress THCAS with Ma_OvaABC and tCsPT4 as the next step.

THCAS has been demonstrated to cyclize prenylated OA analogs with varying alkyl chain lengths, including CBGA (C5) and CBGVA (C3) (Luo et al., 2019). Upon introducing plasmid expressing THCAS targeted to the vacuole of JHY686 (Zirpel et al., 2017), tCsPT4, and Ma_OvaABC in JHY686, selected ion monitoring of *m/z* [M-H]^−^ 385 led to the appearance of trace amounts of a compound that could be ∆^9^–THCPA (Fig. 4). The low titer (estimated to be <100 μg/L) hindered isolation of the compound for complete NMR analysis.

### Improving ∆^9^–THCPA Production From Yeast and Nonenzymatic Decarboxylation to ∆^9^–THCP

**Fig. 5.**
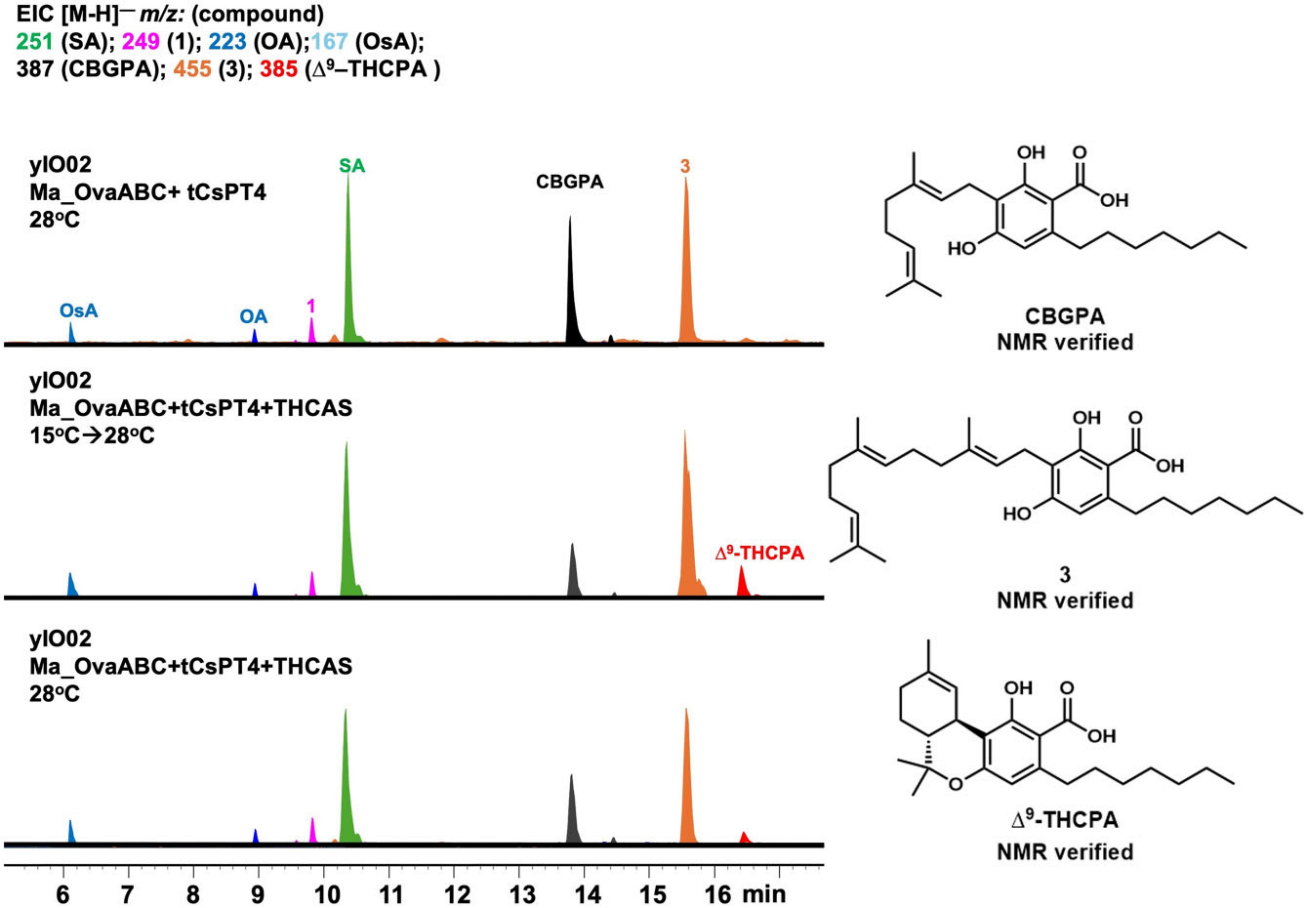
Metabolic analysis of heterologous expression of Ma_OvaABC, tCsPT4, and THCAS in yIO02 strain that has elevated intracellular GPP levels. The optimal condition is when the cells were initially cultured at 15°C for 3 days, followed by an incubation at 28°C for an additional 4 days. The titer of ∆^9^–THCPA reached ∼5 mg/L under this condition. **3**, which is the farnesylated analog of CBGPA remains a major shunt product under all conditions.

Examining the distribution of products from the yeast strain that expresses all enzymes, it is clear that geranylation of SA to CBGPA, and the oxidative cyclization of CBGPA to ∆^9^–THCPA, are both highly inefficient in yeast. To address the first issue, we hypothesized that the free GPP pool in yeast is not sufficient to support high titer CBGPA formation. The low intracellular GPP concentration in yeast is well documented, and numerous approaches have been developed (Chen et al., 2020). Based on these approaches, we first integrated additional copies of genes encoding key enzymes from the mevalonate pathway into the yeast chromosome, including those encoding truncated 3-hydroxy-3-methylglutaryl-coenzymeA reductase (tHMG1) and isopentenyl-diphosphate delta isomerase 1 (IDI1). Furthermore, the mutated FPP synthases from *S. cerevisiae* (ERG20*K197G) and the *Gallus gallus* (mFPS*N144W) were integrated to increase GPP accumulation, and to arrive at the strain yIO02 (for detailed strain genotype, see Table S3) (Yee et al., 2019). The final strain yIO02 retained the endogenous FPP synthase, *ERG20* gene. Co-expression of tCsPT4 with Ma_OvaABC in yIO02 resulted in increased titer of CBGPA (∼30 mg/L) (Fig. 5). However, a substantial increase in the titer of the FPP-derived shunt product **3** (∼150 mg/L) was also observed (Figs 4 and S4), suggesting this shunt pathway diverts the flux of the desired pathway. Subsequently, co-fexpression of tCsPT4 and THCAS with Ma_OvaABC in yIO02 resulted in an improved titer (∼1 mg/L) of the compound with MWT of 386 (Fig. 5). This increase in titer enabled production and isolation from larger scale yeast cultures. Complete NMR analysis confirmed that the compound is indeed the expected ∆^9^–THCPA (Table S5, Figs S13–S18).

The low conversion of CBGPA to ∆^9^–THCPA was attributed to the elevated temperature of the yeast cultures, which is typically performed at 28°C. Previous expression of THCAS using *Pichia pastoris* as a host showed the enzyme expressed optimally when expression was induced at 15°C (Zirpel et al., 2015). Therefore, to further increase ∆^9^–THCPA production, culturing conditions were tuned by varying temperature to achieve a balance of yeast metabolic activity, tCsPT4 activity and THCAS activity. The best condition achieved was first culturing the yeast at 15°C for 3 days to allow proper expression and folding of THCAS, followed by increasing the temperature to 28°C for 4 days to elevate the cellular metabolic activity. A 5-fold increase in the titer of ∆^9^–THCPA (∼5 mg/L) was achieved under these conditions, with **3** remaining as the major shunt product (Figs 5 and S4). Keeping the temperature at 15°C for the entire duration of the culture did not lead to the formation of any related products. Low levels of CBGA were detected in the yeast host, with titers of ∼1 mg/L (Fig. S2), while no detectable production of ∆^9^–THCA was observed.

### Nonenzymatic Decarboxylation of ∆^9^–THCP

The conversion of ∆^9^–THCPA to the potent cannabinoid ∆^9^–THCP involves thermal decarboxylation that is nonenzymatic. By drying the purified ∆^9^–THCPA sample and heating at 130°C for 1 hr, >90% conversion to ∆^9^–THCP was achieved (Wang et al., 2016). The final product was structurally verified by NMR characterization (Table S6, Figs S19–S24). ∆^9^–THCP was first isolated from the *Cannabis FM2 strain* in 2019 and was demonstrated to have 30 times higher affinity to the human CB_1_ receptor and 5–10 times higher affinity to the human CB_2_ receptor, when compared to those of ∆^9^–THC (Citti et al., 2019). Our microbial platform, with minimal optimization, is able to produce the immediate precursor to this molecule at ∼5 mg/L, compared to the ∼0.13 mg/g of ∆^9^–THCP isolated from the producing plant.

### Structural Modeling to Understand Prenyl Donor Promiscuity of CsPT4

**Fig. 6.**
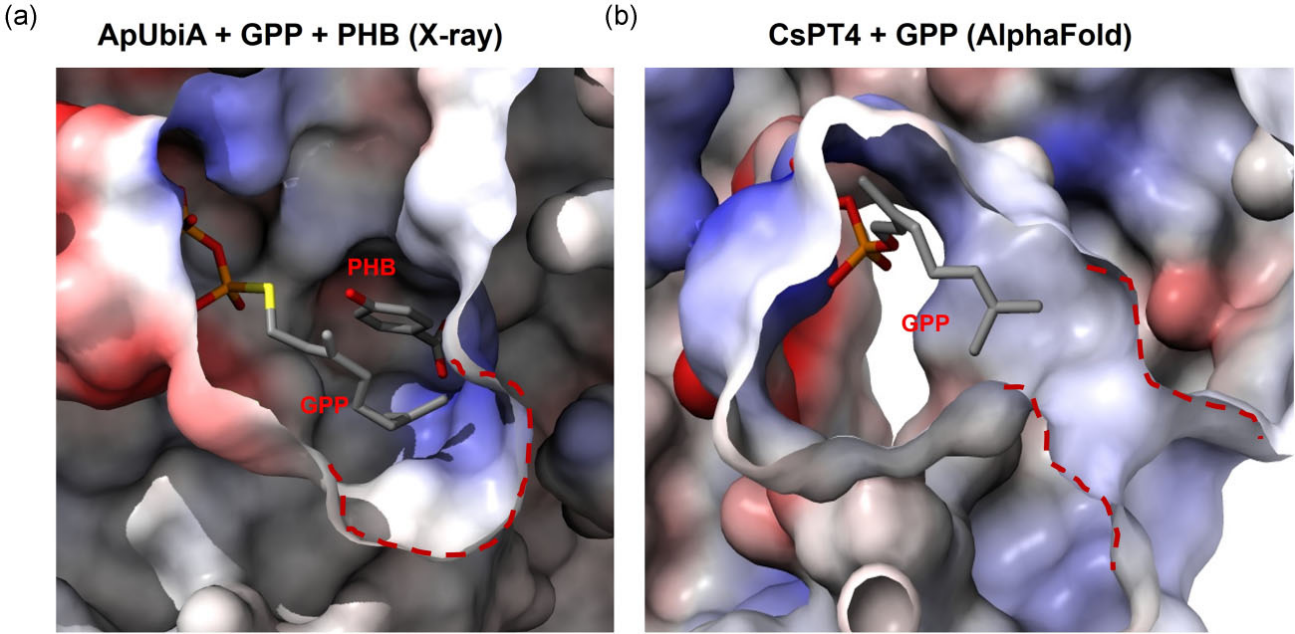
Electrostatic representation of the binding pockets of (a) ApUbiA (PDB: 4OD5) and (b) CsPT4. The CsPT4 structure was predicted by Alphafold 3, and docking was performed by replica-exchange Monte Carlo simulation. (Zhang et al., 2020)

The accumulation of shunt product **3** with the farnesyl group indicates CsPT4 is promiscuous towards the prenyl donors, which is consistent with previous reports (Tanaya et al., 2023). Given FPP is more abundant in yeast and **3** is a dead-end product, such promiscuity depletes the pool of SA that can be geranylated for the biosynthesis of CBGPA and ultimately ∆^9^–THCPA. It should be noted, however, C5-farnesyl-CBGA exhibits antineuroinflammatory and antibacterial activities (Yan et al., 2025), suggesting that **3** could also exhibit potential bioactivities. In order to understand the molecular basis for substrate recognition, we performed structural analysis of tCsPT4 using Alphafold 3 accompanied by docking with the GPP substrate and comparison to reported UbiA-prenyltransferase structures (Abramson et al., 2024). The archaeal membrane-bound UbiA prenyltransferase (PDB: 4OD4) was crystalized with *p*-hydroxybenzoate and GPP (Cheng & Li, 2014). The crystal structure revealed a large and hydrophobic active site where GPP was cocrystalized, providing a structural basis to the observed prenyl donor promiscuity (Fig. 6a). Similarly, the predicted structural prediction of tCsPT4 revealed a similar, large hydrophobic cavity. The binding pocket of CsPT4 comprises of 2 distinct parts: one region is rich in aspartic acid which can bind phosphate and Mg^2+^; and a small opening which is the prenyl-binding chamber. The Alphafold predicted structure and docking results indicate that tCsPT4 could indeed accommodate longer prenyl substrates such as FPP (Figs 6b and Fig. S4). Comprehensive mutation screening of the prenyl binding pocket can be deployed to identify tCsPT4 mutants that can exclude FPP in favor of GPP. Recently, a promiscuous prenyltransferase AscC was engineered to display improved prenyl donor selectivity through site-directed mutagenesis (Yan et al., 2025). Based on the predicted binding pocket of tCsPT4, 3 residues—A149, G146, and V39—were identified as potentially influencing the size of the substrate-binding chamber. Site-directed mutagenesis was performed to replace these residues with bulkier amino acids. However, these mutations significantly impaired the prenylation efficiency of tCsPT4 based on yeast-based product assay (Fig. S6). Hence, additional directed mutagenesis and protein engineering efforts are required to suppress FPP recognition.

### Genome Mining for a Fungal Prenyltransferase With OsA as Substrate

Expression of Ma_OvaABC in yIO02 and JHY686 resulted in the production of a compound with MWT and UV absorbance consistent with that of OsA (Fig. 4). The biosynthesis of OsA by Ma_OvaABC in yeast was unexpected, as this compound was not observed in *A. nidulans* (Fig. 3b). OsA is a 2,4-dihydroxybenzoic acid with a C1 substitution, which biosynthetically can be synthesized from the NRPKS after priming by acetyl-CoA. We therefore attribute the formation of OsA to the direct priming of Ma_OvaB in yeast and bypassing the octanoyl starter unit provided by the HRPKS Ma_OvaA. Nevertheless, the formation of OsA can be exploited for the biosynthesis of the corresponding C1 analog of ∆^9^–THCA, ∆^9^–THCCA. The decarboxylated product of ∆^9^–THCCA, ∆^9^–THCC, is a very rare cannabinoid isolated from *Cannabis sativa L*. Unlike most other cannabinoids that bind to CB_1_ and CB_2_ receptors, ∆^9^ –THCC has negligible affinity to CB_1_ and CB_2_ but functions as an activator of the TRPA1 calcium channel which plays an integral role in pain perception (Ross et al., 2005; Andersson et al., 2011).

**Fig. 7.**
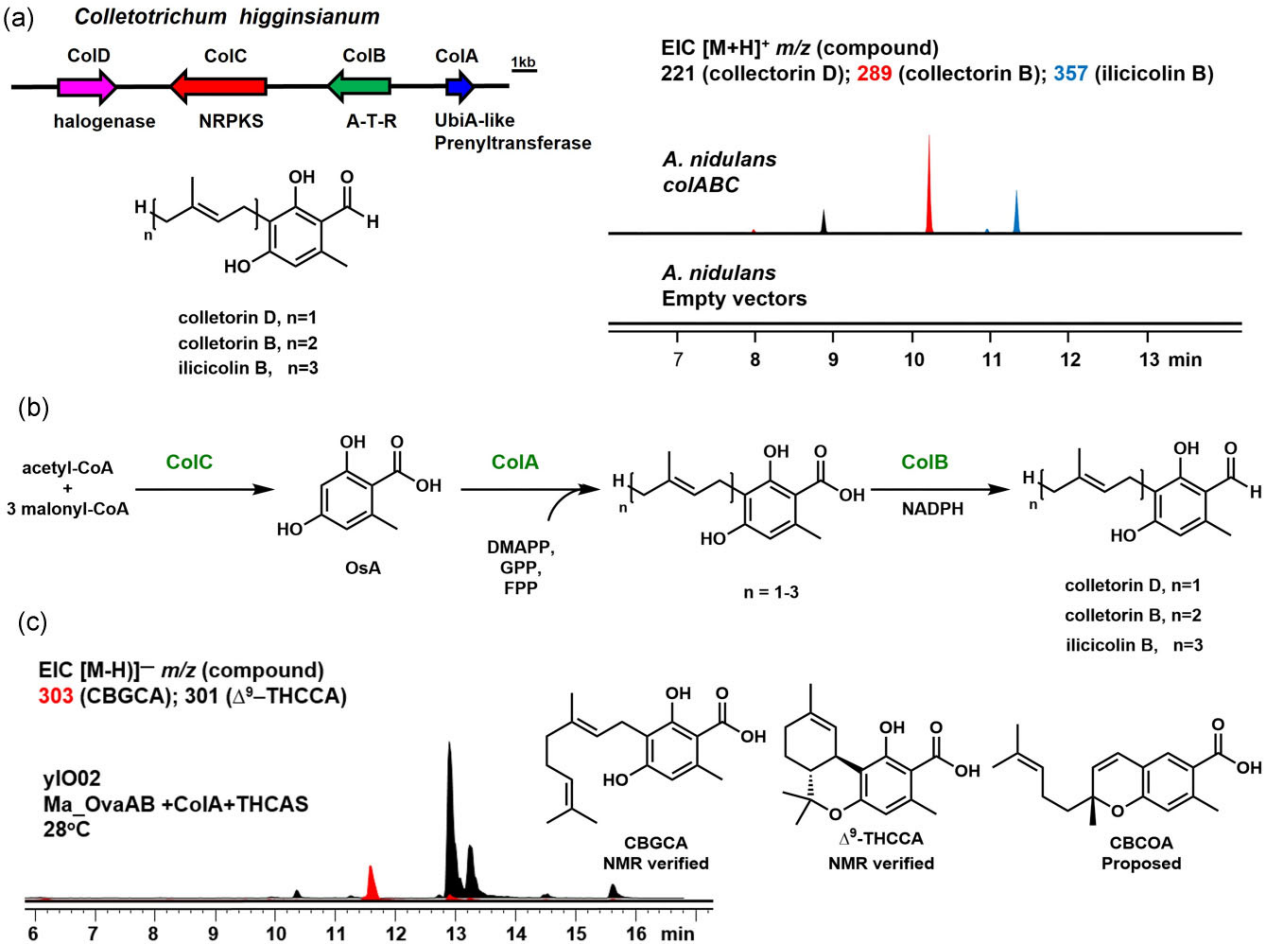
Discovery of the fungal UbiA prenyltransferase, ColA, and application for biosynthesis of THCCA. (a) The fungal biosynthetic gene cluster containing ColA, and metabolic analysis of heterologous expression of *col* gene cluster in *A. nidulans*. (ATR domains: adenylation-thiolation-reductase). The control trace is the metabolic analysis of the *A. nidulans* expressing empty vectors. From the metabolic analysis of *A. nidulans* expressing genes *colABC*, the transformation produced compounds with equivalent MWT to colletorin D, colletorin B and ilicicolin B. (b) The proposed biosynthetic pathway of the colletorin products; (c) Metabolic analysis of heterologous expression of ColA with Ma_OvaABC and THCAS in yIO02. Two new metabolites with MWT 304 and 302 are produced, which were NMR verified to be CBGCA and ∆^9^–THCCA, respectively. A third metabolite with MWT of 302 is proposed to be CBGOA.

In the yeast transformant that produced CBGPA (Fig. 4), the geranylated cannabigerorcinic acid (CBGCA) and ∆^9^–THCCA were not detected, suggesting tCsPT4 could not prenylate OsA. Hence, an OsA-specific prenyltransferase is required. Based on the lack of function with bacterial prenyltransferases in fungi, we reasoned a fungal prenyltransferase is more likely to be functionally expressed in yeast. With this in mind, we sought to identify previously isolated 2,4-dihydroxybenzoic acid fungal metabolites with *C*-prenylation at the C-3 position. A particular group of related prenylated orsellinaldehyde derivatives was particularly attractive with respect to enzyme mining (Fig. 7a). A number of such derivatives are reported from various plant pathogens, but in particular, the colletorins and chlorinated colletochlorins isolated from *Colletotrichum* species of the Destructivum complex (*C. nicotinae, C. destructivum, C. higginsianum*) were notable in that in multiple accounts these organisms produced major isolated products that are *C*-geranylated (Ellestad et al., 1969; Kosuge et al., 1974a, 1974b; Sasaki et al., 1974). Based on these reports, the prenyltransferase involved in the biosynthesis of the colletorins and colletochlorins can be a potential candidate for predominantly geranylating the C-3 position amongst a diverse prenyl-donor pool.

To identify the desired colletorin BGC harboring our target prenyltransferase, we first set out to define a minimum BGC based on the structural constituents of the colletorins and structurally related products. The proposed pathway includes formation of the OSA core by an NRPKS, which is modified by an ensemble of a prenyltransferase, a halogenase, and a reductase. Searching available *Colletotrichum* genomes within the Destructivum complex (namely *C. destructivum and C. higginsianum*) resulted in only one such BGC that meets these criteria, which we named as the *col* BGC (Fig. 7a). The *col* BGC is comprised of genes that encode an NRPKS (*colC*), an NRPS-like enzyme with the domain architecture adenylation (A)-thiolation (T)-reductase (R) (*colB*), a halogenase (*colD*), and an UbiA-prenyltransferase (*colA*) (Fig. 7a). The proposed sequence of reactions catalyzed by these enzymes is shown in Fig. 7b.

To test our hypothesis, we heterologously expressed the *col* cluster in *A. nidulans*. The transformant expressing the ColB, ColC, and the prenyltransferase ColA produced compounds with the same MWTs of colletorin D, colletorin B, and ilicicolin B (Fig. 7a), at a ratio of 1:5:2. In terms of the relative retention time and UV absorbance, we proposed that the 3 new compounds are the colletorin compounds with different prenyl groups. These heterologous expression results indicated that WT ColA could not only effectively prenylate OsA but also yielded more of the desired geranylated product compared to the farnesylated compound. To determine if ColA can be integrated into the yeast strain to produce ∆^9^–THCCA, ColA was coexpressed with Ma_OvaABC and THCAS in yIO02 (Fig. 7c). The yeast transformant was then cultured with the temperature shift condition previously detailed. Two new compounds with MWT of 304 and 302, which match that of CBGCA and ∆^9^–THCCA, respectively, were found in the extracts. Large-scale yeast culture was performed, followed by purification of the compounds. Complete NMR analysis confirmed the two compounds are indeed CBGCA (Table S8, Figs S31–S36) and ∆^9^–THCCA (∼16 mg/L) (Table S9, Fig. S4 and S37–S42). An additional metabolite with MWT 302 was also identified in addition to the major product ∆^9^–THCCA (Fig. 7c). This minor product was proposed to be cannabichromeorcinic acid (CBCOA) based on the production of CBCA in addition to ∆^9^–THC from CBGA catalyzed by THCAS (Zirpel et al., 2018).

## Conclusion

In summary, we have engineered *S. cerevisiae* to achieve *de novo* biosynthesis of two rare cannabinoids, ∆^9^–THCPA (∼5 mg/L) and ∆^9^–THCCA (∼16 mg/L). The engineered pathways include fungal enzymes for biosynthesis of the 2,4-dihydroxy-6-alkylbenzoic acid core; either a plant or a fungal prenyltransferase for regioselective prenylation; and the plant THCAS for oxidative cyclization to the cannabinoid. The titers were improved with increased GPP production and optimized temperature conditions. There remains significant room for further optimization, especially with regard to the prenyl donor promiscuity of tCsPT4. Our work demonstrates that this yeast platform can be flexible in combining different enzymes to access rare cannabinoids. With the diversity of fungal BGCs capable of producing 2,4-dihydroxylbenzoic acid or resorcylic acid natural products, additional rare cannabinoids can be produced, especially those containing different alkyl chains.

## Methods

### Plasmids and Strains

*E. coli* TOP10 was used for cloning, following standard recombinant DNA techniques. DNA restriction enzymes were used as recommended from the manufacturer (New England Biolabs, NEB). Q5 High Fidelity DNA polymerase (NEB) is used for all PCR reactions. The plasmids were sequenced by Laragen and Primordium Lab. Gene integrations and deletions were performed through CRISPR-Cas9 unless otherwise noted using the protocol described by Horwitz et al. (2015). Guide RNA sequences were designed with the CRISPRy Cas9 target finder (Jakočiūnas et al., 2015). Plasmids with gRNA sequences contained *hygR* or *kanMX* antibiotic resistance markers. The primers used in this study are listed in Table S1. Plasmids used for cloning are listed in Table S2. Strain genotypes are listed in Table S3. Knockouts were performed using antibiotic markers. To remove GES from previous engineered strain S12 (Table S3), a hygromycin marker flanked with 30–60 bp homology to CrGES was transformed into the S12 strain to remove CrGES. The knockout was confirmed by plating on selection plate containing hygromycin and by sequencing the knockout region. A zeocin marker flanked with 30–60 bp homology to ObGES was transformed into knockout ObGES. The double knockout strain was confirmed by plating on plates containing hygromycin and zeocin and by sequencing of both knockout regions.

Three plasmid vectors, pYTU, pYTP, and pYTR containing auxotrophic markers for uracil (*pyrG*), pyridoxine (*pyroA*), and riboflavin (*riboB*) respectively were used to construct plasmids for *A. nidulans* heterologous expression. *gpdA* promoters from *Penicillium oxalicum* (constitutive *POgpdA*)*, A. niger* (constitutive *gpdA, glaA* induced by starch) and *Penicillium expansum* (constitutive *PEgpdA*) were amplified by PCR. pYTP and pYTR were digested with *PacI*/*NotI*. pYTU was digested with *PacI*/*NotI* (keep *glaA* on vector) or *PShAI*/*NotI* (abolish glaA from vector). The amplified gene fragments and the corresponding vectors were co-transformed into *S. cerevisiae* JHY651 for homologous recombination. The yeast plasmids were extracted using Zymoprep^TM^ Yeast Plasmid Miniprep I (Zymo Inc. USA), and then electrically transformed into *E. coli* TOP10 to isolate single plasmids. The plasmids were extracted from *E. coli* using the Zyppy^TM^ Plasmid Miniprep Kit (Zymo Research).

### Yeast Culturing for Metabolite Production

Single colonies of each strain were inoculated in 1 mL YPD. For plasmid-bearing strains, single colony transformants were inoculated in 1 mL synthetic defined (SD) 2% glucose media with the appropriate dropouts. Starter cultures were shaken at 28°C and 250 rpm for 16–24 hr. Culture tubes contained 2 mL of fresh YPD with 100 μL of starter culture. For cannabinoid acid production, the culture was incubated at 15°C, 220 rpm for 3 days and then changed to 28°C, 220 rpm for 4 days. For large-scale extraction, a single transformant colony was picked into 1 mL selection media incubated at 28°C, 220 rpm overnight. Then 1 mL seed culture was transferred into 40 mL selection media incubated at 28°C, 220 rpm overnight. Then 40 mL seed culture was transferred into 800 mL YPD for isolation of the products. For THCPA production, the culture was incubated at 15°C, 220 rpm for 3 days and then changed to 28°C, 220 rpm for 4 days.

### Culture Extraction and Quantification, and Structural Determination

For small-scale yeast culture extraction, 300 μL culture sample was extracted with 300 μL organic phase consisting of 25% acetone and 75% ethyl acetate with 0.1% FA 3 times. The samples were vortexed for 1 min, then centrifuged for 10 min. All the extracts were dried, and the residues were dissolved in MeOH for analysis. LC-MS analyses were performed on a Shimadzu 2020 EV LC-MS with a reverse phase column (Phenomenex Kinetex 1.7 µm C18 100 Å, LC Column 100 × 2.1 mm) using positive-and negative-mode electrospray ionization with a linear gradient of 5–95% CH_3_CN-H_2_O with 0.1% formic acid (v/v) in 15 min followed by 95% CH_3_CN for 3 min with a flow rate of 0.3 ml/min and a 6545 Q-TOF high resolution mass spectrometer (UCLA Molecular Instrumentation Center) using the solvent program (1% CH_3_CN-H_2_O 2 min, then 1–95% CH_3_CN-H_2_O (both with 0.1% formic acid, v/v) in 9 min followed by 95% CH_3_CN-H_2_O for 6 min at a flow rate of 0.8 mL/min).

To isolate sufficient amount of compound for NMR analysis, large-scale yeast culture was performed, and the cells were separated by centrifugation. The cell pellet was extracted by acetone and the supernatant was extracted by EtOAc. The extracts were dried, combined and then redissolved in 15 mL methanol. The insoluble impurities were discarded. Ten gram of celite was added to the mixture and methanol was evaporated. The dried crude was purified with the CombiFlash system (Teledyne) using reverse phase gradient elution with water (A) and acetonitrile (B) with 0.1% FA. The fractions containing the desired product were dried and then redissolved in methanol. Semipreparative HPLC was performed on an S7 UltiMateTM 3000 Semi-Preparative HPLC (ThermoFisher) using a COMOSIL 5C18-AR-II (5 µm, 4.6 × 250 mm) or COSMOSIL 5C18-MS-II column (5 µm, 250 × 10 mm). The flow rate for HPLC was set at 3 mL/min. NMR spectra were obtained with a Bruker AV500 spectrometer with a 5 mm dual cryoprobe at the UCLA Molecular Instrumentation Center (^1^H NMR 500 MHz, ^13^C NMR 125 MHz).

Quantification of the compounds was performed by first making a standard curve on the HPLC. Known concentrations of isolated compounds were analyzed on the HPLC (ThermoFisher), and a standard curve was constructed correlating the area under the UV peak corresponding to the compound to the concentration of the compound. Cultured samples were then extracted and analyzed on the HPLC, where the area under the peak was used to calculate the concentration of the sample.

## Supplementary Material

kuaf013_Supplemental_File

## Data Availability

Data available on request.
