# Supplementary material for: Microbial biosynthesis of rare cannabinoids
Source: J Ind Microbiol Biotechnol. 2025 May 13;52:kuaf013. doi: 10.1093/jimb/kuaf013 (PMC12134893; doi:10.1093/jimb/kuaf013)
Supplement: kuaf013_Supplemental_File [file kuaf013_supplemental_file.pdf]

Supporting Information

Microbial Biosynthesis of Rare Cannabinoids

Chunsheng Yan,[1] Ikechukwu C. Okorafor,[1,3]* Colin W. Johnson,[2] Kendall N. Houk,[2] Neil K. Garg,[2] Yi Tang [1,2]*

[1] Department of Chemical and Biomolecular Engineering, [2] Department of Chemistry and Biochemistry, [3] California NanoSystems Institute, University of California, Los Angeles, CA 90095, USA.

Table of Contents

Experimental procedures 5

1. **Strains and culture conditions** 5

2. **Heterologous expression of the gene cluster in *A. nidulans*** 5

Supplementary Tables 6

Table S1. Primers used in this study.. 6

Table S2. Plasmids used in this study.. 7

Table S3. Yeast Strains Used in This Study [2,3,4,5].. 8

Table S4. ^{1}H (500 MHz) and ^{13}C NMR (125 MHz) for CBGPA in DMSO-d_6 9

Table S5. ^{1}H (500 MHz) and ^{13}C NMR (125 MHz) for Δ^9–THCPA in DMSO-d_6.. 10

Table S6. ^{1}H (500 MHz) and ^{13}C NMR (125 MHz) for Δ^9–THCP in CD_3OD 11

Table S7. ^{1}H (500 MHz) and ^{13}C NMR (125 MHz) for **3** in DMSO-d_6.. 12

Table S8. ^{1}H (500 MHz) and ^{13}C NMR (125 MHz) for CBGCA in CD_3OD.. 13

Table S9. ^{1}H (500 MHz) and ^{13}C NMR (125 MHz) for Δ^9–THCCA in CD_3OD 14

Supplementary Figures.. 15

Figure S1. Previous work on microbial production of cannabinoids.. 15

Figure S2. Localization assays of NphB in *A. nidulans*.. 16

Figure S3. Standard curves of CBGPA, shunt product **3**, SA, Δ^9–THCPA,Δ^9–THCCA, and CBGA.. 17

Figure S4. Metabolite analysis of ylO02 transformant at different temperatures. 18

Figure S5. The structural comparison between three UbiA-prenyltransferases, 4OD4, CsPT4 and ColA. 19

Figure S6. Metabolite analysis of ylO02 transformant expressing tCsPT4 mutatnts.. 20

Figure S7 ^{1}H NMR spectrum of compound CBGPA in DMSO-d_6 (500 MHz).. 21

Figure S8 ^{13}C NMR spectrum of compound CBGPA in DMSO-d_6 (125 MHz).. 22

Figure S9 ^{1}H-^{13}C HSQC spectrum of compound CBGPA in DMSO-d_6 (500 MHz).. 23

Figure S10 ^{1}H-^{13}C HMBC spectrum of compound CBGPA in DMSO-d_6 (500 MHz).. 24

.... 25

Figure S11 ^{1}H-^{1}H COSY spectrum of compound CBGPA in DMSO- d_6 (500 MHz)..........25
Figure S12 ^{1}H-^{1}H NOESY spectrum of compound CBGPA in DMSO-d_6 (500 MHz).........26
Figure S13 ^{1}H NMR spectrum of compound Δ^9–THCPA in DMSO-d_6 (500 MHz) 27
Figure S14 ^{13}C NMR spectrum of compound Δ^9–THCPA in DMSO-d_6 (125 MHz) 28
Figure S15 ^{1}H-^{13}C HSQC spectrum of compound Δ^9–THCPA in DMSO-d_6 (500 MHz) 29
Figure S16 ^{1}H-^{13}C HMBC spectrum of compound Δ^9–THCPA in DMSO-d_6 (500 MHz).....30
Figure S17 ^{1}H-^{1}H COSY spectrum of compound Δ^9–THCPA in DMSO-d_6 (500 MHz).......31
Figure S18 ^{1}H-^{1}H NOESY spectrum of compound Δ^9–THCPA in DMSO-d_6 (500 MHz).....32
Figure S19 ^{1}H NMR spectrum of compound Δ^9–THCP in CD_3OD (500 MHz).................33
Figure S20 ^{13}C NMR spectrum of compound Δ9–THCP in CD3OD (125 MHz) **.............**34
Figure S21 ^{1}H-^{13}C HSQC spectrum of compound Δ^9–THCP in CD_3OD (500 MHz)..........35
Figure S22 ^{1}H-^{13}C HMBC spectrum of compound Δ^9–THCP in CD_3OD (500 MHz) 36
Figure S23 ^{1}H-^{1}H COSY spectrum of compound Δ^9–THCP in CD_3OD (500 MHz) 37
Figure S24 ^{1}H-^{1}H NOESY spectrum of compound Δ^9–THCP in CD_3OD (500 MHz) 38
Figure S25 ^{1}H NMR spectrum of compound **3** in DMSO-d_6 (500 MHz) 39
Figure S26 ^{13}C NMR spectrum of compound **3** in DMSO-d_6 (125 MHz) 40
Figure S27 ^{1}H-^{13}C HSQC spectrum of compound **3** in DMSO-d_6 (500 MHz) 41
Figure S28 ^{1}H-^{13}C HMBC spectrum of compound **3** in DMSO-d_6 (500 MHz).................42
Figure S29 ^{1}H-^{1}H COSY spectrum of compound **3** in DMSO-d_6 (500 MHz)...................43
....44
Figure S30 1H-1H NOESY spectrum of compound **3** in DMSO- d_6 (500 MHz)...............44
Figure S31 ^{1}H NMR spectrum of compound CBGCA in CD_3OD (500 MHz) 45
Figure S32 ^{13}C NMR spectrum of compound CBGCA in CD_3OD (125 MHz) 46
Figure S33 ^{1}H-^{13}C HSQC spectrum of compound CBGCA in CD_3OD (500 MHz) 47
Figure S34 ^{1}H-^{13}C HMBC spectrum of compound CBGCA in CD_3OD (500 MHz)...........48
Figure S35 ^{1}H-^{1}H COSY spectrum of compound CBGCA in CD_3OD (500 MHz).............49
Figure S36 ^{1}H-^{1}H NOESY spectrum of compound CBGCA in CD_3OD (500 MHz)...........50
Figure S37 ^{1}H NMR spectrum of compound Δ^9–THCCA in CD_3OD (500 MHz)...............51
Figure S38 ^{13}C NMR spectrum of compound Δ^9–THCCA in CD_3OD (125 MHz).............52

Figure S39 ^{1}H-^{13}C HSQC spectrum of compound Δ^9–THCCA in CD_3OD (500 MHz) 53
Figure S40 ^{1}H-^{13}C HMBC spectrum of compound Δ^9–THCCA in CD_3OD (500 MHz) 54
Figure S41 ^{1}H-^{1}H COSY spectrum of compound Δ^9–THCCA in CD_3OD (500 MHz) 55
Figure S42 ^{1}H-^{1}H NOESY spectrum of compound Δ^9–THCCA in CD_3OD (500 MHz) 56
References 57

Experimental procedures

1. **Strains and culture conditions**

Aspergillus nidulans (*A. nidulans*) A1145 ΔSTΔEM was grown at 28 °C on CD agar (1 L: 10 g glucose, 50 mL of 20X nitrate salts, 1 mL of trace elements, 20 g agar) or CDST agar (if use *glaA* promoter, 20 g starch, 20 g casamino acids (acidic digest), 50 mL 20X nitrate salts, 1 mL trace elements, 20 g agar) for heterologous expression of the gene cluster. The 20X nitrate salts are prepared as: 120 g of $NaNO_3$, 10.4 g of KCl, 10.4 g of $MgSO_4$•$7H_2O$, 30.4 g of KH_2PO_4 dissolved in 1 L distilled water. The trace element solution was prepared as: 2.20 g of $ZnSO_4$•$7H_2O$, 1.10 g of H_3BO_3, 0.50 g of $MnCl_2$•$4H_2O$, 0.16 g of $FeSO_4$•$7H_2O$, 0.16 g of $CoCl_2$•$5H_2O$, 0.16 g of $CuSO_4$•$5H_2O$, and 0.11 g of $(NH_4)_6Mo_7O_{24}$•$4H_2O$ dissolved in 100 mL of distilled water, and the pH was adjusted to 6.5. All *Escherichia coli* strains were culture in LB media with carbenicillin antibiotic. Yeast strains were culture in YPD media (yeast extract 1%, peptone 2%, glucose 2%) at 220 rpm, 28 °C/15 °C.

2. **Heterologous expression of the gene cluster in *A. nidulans***

To prepare protoplasts, *A. nidulans* A1145 ΔSTΔEM was grown Oatmeal agar plates supplemented with 10 mM of uridine, 5 mM of uracil, 0.5 µg/mL of pyridoxine HCl and 2.5 µg/mL of riboflavin at 37 °C for 4 days. Fresh spores of *A. nidulans* A1145 ΔSTΔEM were inoculated into 25 mL of liquid CD media supplemented with 10 mM of uridine, 5 mM of uracil, 0.5 µg/mL of pyridoxine HCl and 2.5 µg/mL of riboflavin in a 125 mL flask and germinated at 28 °C 250 rpm for 16 hours. Mycelia were harvested by centrifugation at 4000 rpm for 20 min and washed with 10 mL of osmotic buffer (1.2 M of $MgSO_4$, 10 mM of sodium phosphate, pH 5.8). The mycelia were transferred into 10 mL of osmotic buffer containing 30 mg of lysing enzymes from *Trichoderma* and 20 mg of Yatalase in a 125 mL flask. The cells were digested for 5 hours at 37 °C, 80 rpm. Cells were harvested in a 50 mL falcon tube and gently overlaid with 10 mL of trapping buffer (0.6 M of sorbitol, 0.1 M of Tris-HCl, pH 7.0). The cells were then centrifuged at 4300 rpm for 30 min at 4°C, the protoplasts were collected at the interface of the two buffers. The protoplasts were transferred to a sterile 15 mL falcon tube and washed with 3 volumes of STC buffer (1.2 M of sorbitol, 10 mM of $CaCl_2$, 10 mM of Tris-HCl, pH 7.5). After centrifugation at 4300 rpm, 20 min at 4 °C, the supernatant was discarded, and the protoplast pellet was resuspended in 1 mL of STC buffer.

For each transformation, 3 µL of each plasmid (>100 ng/µL) was added to 60 µL of the *A. nidulans* A1145 ΔSTΔEM protoplast suspension prepared as above, and the mixture was incubated for 1 hour on ice. 600 µL PEG solution (60% PEG, 50 mM of $CaCl_2$, and 50 mM of Tris-HCl, pH 7.5) was added to the protoplast mixture, followed by additional incubation at room temperature for 20 min. The mixture was spread on the CD sorbitol plate (CD solid medium with 1.2 M sorbitol and the appropriate supplements: 10 mM of uridine, 5 mM of uracil, 0.5 µg/mL of pyridoxine HCl, and/or 2.5 µg/mL of riboflavin according to the markers in the transformed plasmids) and incubated at 37 °C for 3-4 days.[1]

Supplementary Tables

Table S1. Primers used in this study.

Primer Name	Sequence (5'→3')
HRPKS-F-ADH2P	caactatcaactattaactatatcgtaatatgcaagcgccagcaccatcaagagacg
HRPKS-R-ADH2T	catacttgataatgaaaactataaatcgctagttcaatttcaccaaagtagacatggatg
Ma_OvaB-F-ADH2p	tatcaactattaactatatcgtaataccatatgaaactgcgtgtcgcaaacttc
NRPKS-R-Spg5t	ggtaatagcgcgatgaaacaacgtctttgcctaccctaccgccgcaatgactg
jb SPG5t F	gcaaagacgttgtttcatcgc
spg5t-R	gcttattttctgccgaattttcatgaagtttttatg
Pck1p-F-spg5t	aacttcatgaaaattcggcagaaaataagcataggaaaaaaccgagcttcctttcatcc
PCK1p R	gttgttattttattatggaataattagttgcgtg
ACPTE-F-PCK1p	caactaattattccataataaaataacaacatggccgtcaccgtgtggcaag
ACPTE-R	tcatgactggctcactcgctg
CsPT477t1-F-ADH2P	aactatcaactattaactatatcgtaatacatgtctgctggctctgaccaaattg
CsPT477t-R-SPG5t	ggtaatagcgcgatgaaacaacgtctttgcttaaataaatacgtagacgaaatactcggc
jb SPG5t F	gcaaagacgttgtttcatcgc
spg5t-R	gcttattttctgccgaattttcatgaagtttttatg
proATHCAS-F-PCK1p	caactaattattccataataaaataacaacatgattttcgatgggaccacgatgtc
proAtrunTHCAS-R-CYC1	aatgtaagcgtgacataactaattacatgagtgatgatgagggggcaaaggc
XW55-F-1-cyc1t	tcatgtaattagttatgtcacgcttacattcac
CYC1-R	gcaaattaaagccttcgagcgtcc
TEF1-F-cyc1	gttttgggacgctcgaaggctttaatttgcccgcgaatccttacatcacacc
TEF1p R	tttgtaattaaaacttagattagattgctatgc
Npga-F-TEF1	tagcaatctaatctaagttttaattacaaaatggtgcaagacacatcaagcg
NpgA-R-ADH2Tnew	gataatgaaaactataaatcgtgaaggcatttaggataggcaattacacaccccagtc
colA-XW55-F	actatcaactattaactatatcgtaataccatatggcacctccatcatccaaagtcactg
colA-R-spg5t	ggtaatagcgcgatgaaacaacgtctttgctcaagcggagcctttgagaacg
NphB-F-cox4	tgtagctctagatatctgcttcagggatccatgagcgaggccgctgacg
NphB-F-CyC1	aatgtaagcgtgacataactaattacatgattagtcctccagagaatcgaatgccttg
NphBopt-F-glaA	cttcatccccagcatcattacacctcagcaatgtcagaggcggctgacgtag
NphBopt-R-trpC	tgtttgatgatttcagtaacgttaagtggttaatcttccaggctatcaaacgccttcaac
trpC-F	ccacttaacgttactgaaatcatcaaacagc
trpC-R-pyroA	gagacccaacaaccatgataccaggggaagaaggattacctctaaacaagtgtacctgtg
TislaUbiA-F-POG	gcatacagaacacttcaaacaatcgcaaaaatgccttcaaagacagcgaacaag
TislaUbiA-R-pyroA	gatgagacccaacaaccatgataccaggggaatcggaactgaaacaggagtgttcc
hygr-F-CrGES	atgtctttgccactggctactccattgatcaatgggtaaaaagcctgaactcaccgcgac
hygr-R-CrGES	ttaaaagcatggagtaaagaacagagccttaacgtaattattcctttgccctcggacgag
BleoR-F-ObGES	atgccattatcttcaactcctttgataaatggtgacaactatggccaagttgaccagtgc
BleoR-R-ObGES	ttattgagtgaaaaacaatgcatcgacataattgtctactcagtcctgctcctcggccac
CJ-pYTU-colC_p1-F	attaccccgccacatagacacatctaaacaatgactctcatgccgtccaacac
CJ-pYTU-colC_p1-R	gcatatctctgcaagctctcagctg
CJ-pYTU-colC_p2-F	cgtgtatcaacagctacggtgcttc

CJ-pYTU-colC_p2-R	ggttctggtcatccttgagctctg
CJ-pYTU-colC_p3-F	ctagcagcagcactgacttgacc
CJ-pYTU-colC_p3-R	cacagtggaggacatacccgtaattttctgcagcgatgaaggcaggaaaggag
CJ-pYTP-colA-F	attaccccgccacatagacacatctaaacaatggcacctccatcatccaaagtc
CJ-pYTP-colA-R	gatgagacccaacaaccatgataccaggggcaggagtctgtttctgagcatcagag
CJ-POgpdA-F	tttgctccaggaatacatgtgagcttactg
CJ-POgpdA-R	ttttgcgattgtttgaagtgttctgtatgc
CJ-pYTR-colB-F	attaccccgccacatagacacatctaaacaatgagtgccattactgagcccaag
CJ-pYTR-colB-R	aagggtatcatcgaaagggagtcatccaatggttctgtacatgttgtctatcaggagagg
CJ-pYTU-colC_p2-F	cgtgtatcaacagctacggtgcttc

Table S2. Plasmids used in this study.

Plasmid name	Vector	Genes
pyIO01	XW02	*Ma_OvaA*
pyIO02	XW55	*Ma_OvaBC*
pyIO08	XW55	*TislaUbiA*
pyIO029	Pmd29	*tCsPT4+npgA*
pyIO030	Pmd29	*tCsPT4+npgA+THCAS*
pyIO031	Pmd29	*ColA+npgA+THCAS*
pMetarR01	pYTR	*Ma_OvaA*
pMetarU05	pYTU	*Ma_OvaBC*
pIO10	pYTP	*tCsPT4*
pIO13	pYTP	*NphB*
pIO23	pYTP	*ColA*
pYTU-colC	pYTU	*colC*
pYTP-colB	pYTP	*colB*
pYTR-colA	pYTR	*colA*

Table S3. Yeast Strains Used in This Study [2,3,4,5]

Strain	Parent	Genome modifications to parent	Reference
BY4742	S288C	*MATα his3Δ1 leu2Δ0 ura3Δ0 lys2Δ0*	Brachmann et al. (1998)
DHY214	BY4742	*SAL1*$^{+}$*CAT5(91M) MIP1(661T) MKT1(30G) RME1(INS-308A) TAO3(1493Q) HAP1 +*	Harvey et al. (2018)
JHY651	DHY214	*MATα prb1Δ pep4Δ*	Harvey et al. (2018)
JHY686	JHY651	*ADH2P:npgA:ACS1t*	Harvey et al. (2018)
S1	JHY651	*YPRCTy1–2Δ::iCas9::LEU2*	Yee et al. (2019)
S2	S1	*ura3Δ::TEF1p-CrGES-CYC1t*	Yee et al. (2019)
S3	S2	*rox1Δ::TEF1p-ERG20*(f)ObGES-CYC1t*	Yee et al. (2019)
S4	S3	*oye2Δ::TEF1p-mFPS-CYC1t*	Yee et al. (2019)
S5	S4	*erg9p truncation*	Yee et al. (2019)
S7	S5	*bts1Δ::TEF1p-IDI1-CYC1t*	Yee et al. (2019)
S11	S7	*yjl064wΔ::TEF1p-HMG2*-CYC1t*	Yee et al. (2019)
S12	S11	*ypl062wΔ::GPDp-tHMG1-ADH1t*	Yee et al. (2019)
yIO01	S12	*ura3Δ::TEF1p-Hygr-CYC1t*	*This study*
yIO02	yIO01	*rox1Δ::TEF1p-ERG20*(f)zeo-CYC1t*	*This study*

Table S4. ^{1}H (500 MHz) and ^{13}C NMR (125 MHz) for CBGPA in DMSO-d_6

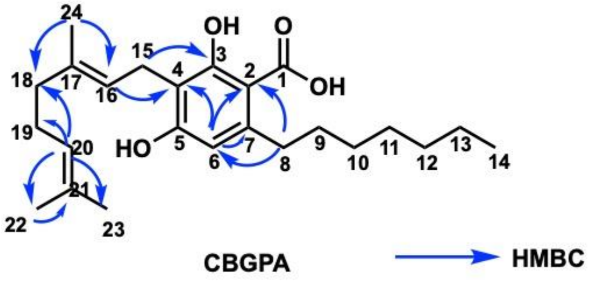

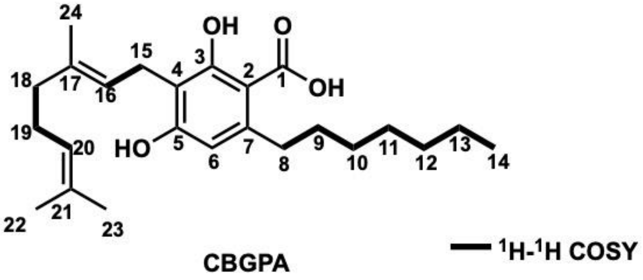

position	^{13}C	^{1}H (J in Hz)
1	173.5	-
2	144.4	-
3	163.2	-
4	111.7	-
5	155.7	-
6	109	6.13 (s, 1H)
7	106.2	-
8	35.6	2.84 (m, 2H)
9	31.6	1.52 (s, 2H)
10	31.4	1.3-1.45 (m, 8H)
11	31.3	
12	30.8	
13	29.3	
14	14	0.86 (t, 3H, 6.7)
15	21.7	3.15 (d, 2H, 7.2)
16	124.2	5.15 (m, 1H)
17	132.6	-
18	40.4	1.9 (m, 2H)
19	28.6	7.99 (q, 2H, 7.4)
20	124.3	5.03 (tq, 1H, 8.6, 1.9)
21	130.6	-
22	26.2	1.59 (m, 3H)
23	17.5	1.52 (m, 3H)
24	15.7	1.69 (m, 3H)

Table S5. ^{1}H (500 MHz) and ^{13}C NMR (125 MHz) for Δ^9–THCPA in DMSO-d_6

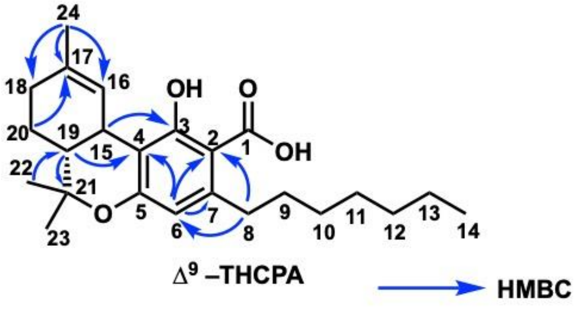

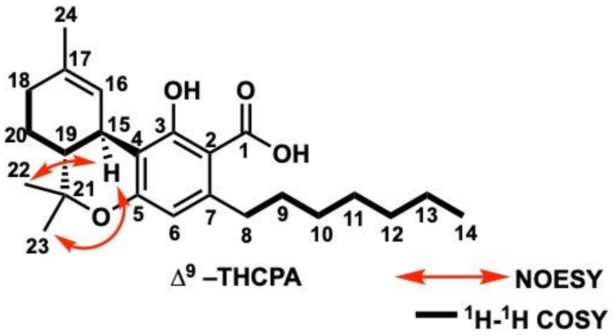

position	^{13}C	^{1}H (J in Hz)
1	172.2	-
2	144.6	-
3	165.6	-
4	110.1	-
5	154.2	-
6	107	5.68 (s, 1H)
7	107.9	-
8	35	1.24 (m, 12H)
9	31.6	
10	31.4	
11	29.5	
12	28.9	
13	22.2	
14	14	0.82 (t, 3H, 6.7)
15	33.5	3 (m, 1H)
16	125.3	6.49 (q, 1H, 1.7)
17	131.1	-
18	30.1	1.24 (m, 2H)
19	27.2	2.05 (m, 2H)
20	45.8	1.49 (s, 1H)
21	76.4	-
22	27.4	1.28 (s, 3H)
23	19.2	0.95 (s, 3H)
24	23.1	1.57 (d,3H, 1.8)

Table S6. ^{1}H (500 MHz) and ^{13}C NMR (125 MHz) for Δ^{9}–THCP in CD_3OD

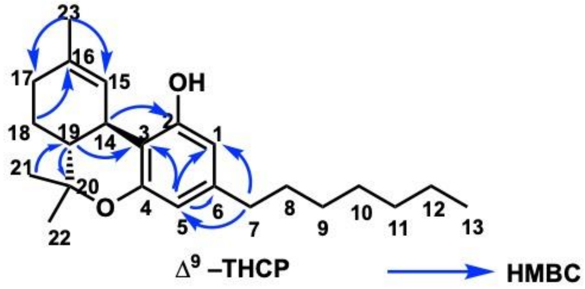

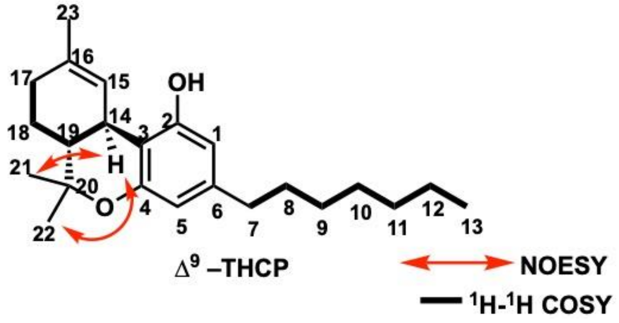

position	^{13}C	^{1}H (J in Hz)
1	108.4	6.16 (s, 1H, 1.8)
2	157.2	-
3	110.4	-
4	155.8	-
5	109.7	5.08 (s, 1H)
6	143.4	-
7	35.2	2.41 (m, 2H)
8	32.4	1.3 (m, 10H)
9	32.3	
10	30.6	
11	30.5	
12	23.7	
13	14.4	0.9 (td, 3H, 7.0, 2.4)
14	33	3.32 (s, 1H)
15	126.2	6.43 (m, 1H)
16	133.5	-
17	30.8	2.32 (t, 2H, 7.5)
18	26.3	1.33 (m, 2H)
19	47.5	1.62 (m, 2H)
20	77.9	-
21	28	1.37 (s, 3H)
22	19.4	1.05 (s, 3H)
23	17.1	1.66 (dd, 3H, 2.3, 1.3)

Table S7. ^{1}H (500 MHz) and ^{13}C NMR (125 MHz) for **3** in DMSO-d_6

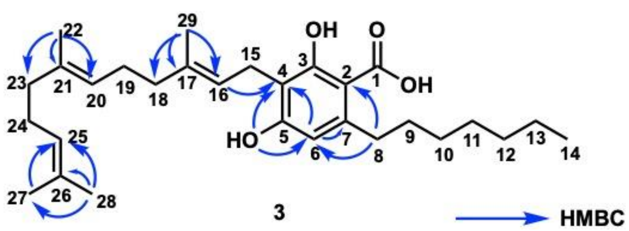

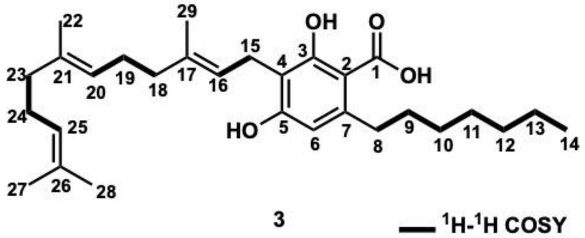

position	^{13}C	^{1}H (*J* in Hz)
1	174.5	-
2	144.4	-
3	164	-
4	112.7	-
5	158.4	-
6	109	6.14 (s, 1H)
7	106.1	-
8	34.6	2.92 (dt, 2H, 12.2, 7.5)
9	31.3	1.63 (m, 2H)
10	31.1	1.42-1.59 (m, 8H)
11	29.4	
12	29.1	
13	29	
14	14	0.85 (m, 3H)
15	21.8	3.06 (d, 2H, 7.3)
16	123.1	5.1 (m, 1H)
17	132.8	-
18	40.3	1.8 (m, 2H)
19	26.3	1.85-2 (m, 2H)
20	124.2	5.05 (m, 1H)
21	134.2	-
22	15.8	1.5 (m, 3H)
23	40.3	1.8 (m, 2H)
24	26.2	1.85-2 (m, 2H)
25	124.1	5.05 (m, 1H)
26	130.5	-
27	25.5	1.63 (m, 3H)
28	15.7	1.53 (m, 3H)
29	17.5	1.54 (m, 3H)
5-OH	-	9.42 (s, 1H)

Table S8. ^{1}H (500 MHz) and ^{13}C NMR (125 MHz) for CBGCA in CD_3OD

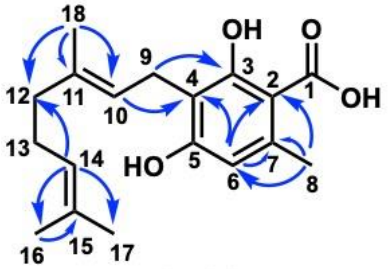

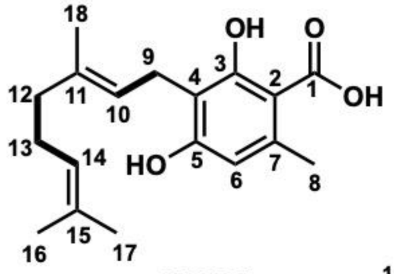

position	^{13}C	^{1}H (J in Hz)
1	ND	-
2	141.7	-
3	164.3	-
4	113.7	-
5	160.5	-
6	111.1	6.17 (s, 1H)
7	106.6	-
8	24.2	2.47 (s, 3H)
9	22.8	3.26 (d, 2H, 7.1)
10	124.1	5.21 (tt, 1H, 5.9, 3.1)
11	134.9	-
12	41	1.94 (dd, 2H, 8.3, 6.7)
13	27.8	2.05 (m, 2H)
14	125.5	5.06 (tdt, 1H, 5.8, 3.0,1.5)
15	132	-
16	25.9	1.62 (m, 3H)
17	17.7	1.52 (m, 3H)
18	16.2	1.75 (d, 3H, 1.3)

ND: The carbon signal was not detected.

Table S9. ^{1}H (500 MHz) and ^{13}C NMR (125 MHz) for Δ^{9}–THCCA in CD_3OD

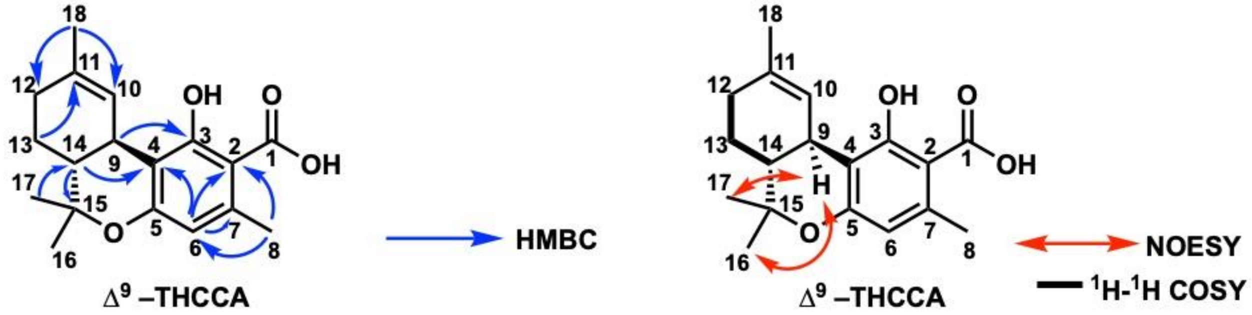

position	^{13}C	^{1}H (*J* in Hz)
1	ND	-
2	141.1	-
3	163.4	-
4	112.1	-
5	157.1	-
6	109	6.09 (s, 1H)
7	111.3	-
8	22.6	2.48 (m, 3H)
9	33.7	3.17 (m, 1H)
10	124.2	6.43 (dt, 1H, 8.4, 1.7)
11	132.3	-
12	30.9	2.11 (d, 2H, 7.8)
13	24.8	1.95 (m, 2H)
14	45.9	1.55 (m, 1H)
15	77.5	-
16	26.4	1.41 (m, 3H)
17	18.2	1.05 (m, 3H)
18	22.1	1.6 (m, 3H)

ND: The carbon signal was not detected.

Supplementary Figures

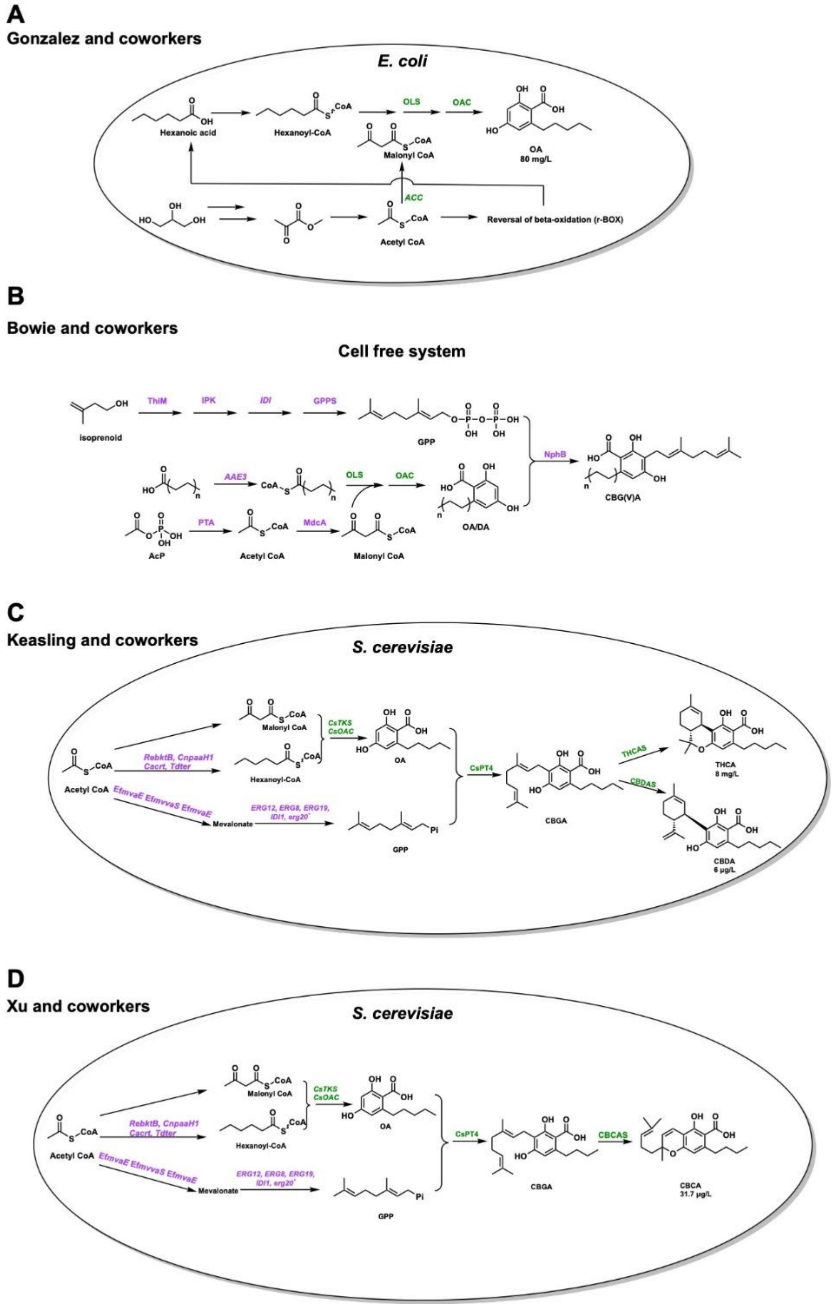

Figure S1. Previous work on microbial production of cannabinoids. A) Gonzalez and co-workers integrated OA biosynthesizing pathway in *E. coli* and achieved 80 mg/L OA production. B) The cell free system designed by Bowie and coworkers relies on *in vitro* reactions to produce CBGA and CBGVA. D) Keasling and co-workers fully integrate the plant pathway in yeast and after engineering the yeast strain, they achieved 8 mg/L Δ^9–THCA production and 4.8 mg/L Δ^9–THCVA production. E) Xu and co-workers also integrated the plant pathway into yeast, and by engineering the yeast strain and expressing CBCAS, they achieved 31.7 µg/L CBCA production.

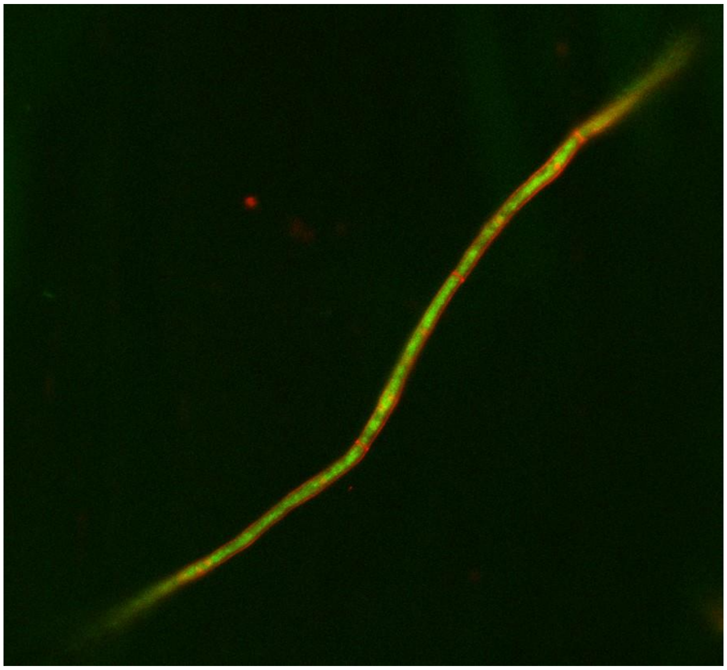

Figure S2. Localization assays of NphB in *A. nidulans*. (146 x 146 μm). The NphBM31s is fused with enhanced green fluorescent protein (EGFP) and stained with Hoescht. The red dots are nucleus. The green signal is from the EGFP fused with NphB. In terms of the picture, the green signal is separated from the nucleus, so the NphB is localized in the cytoplasm.

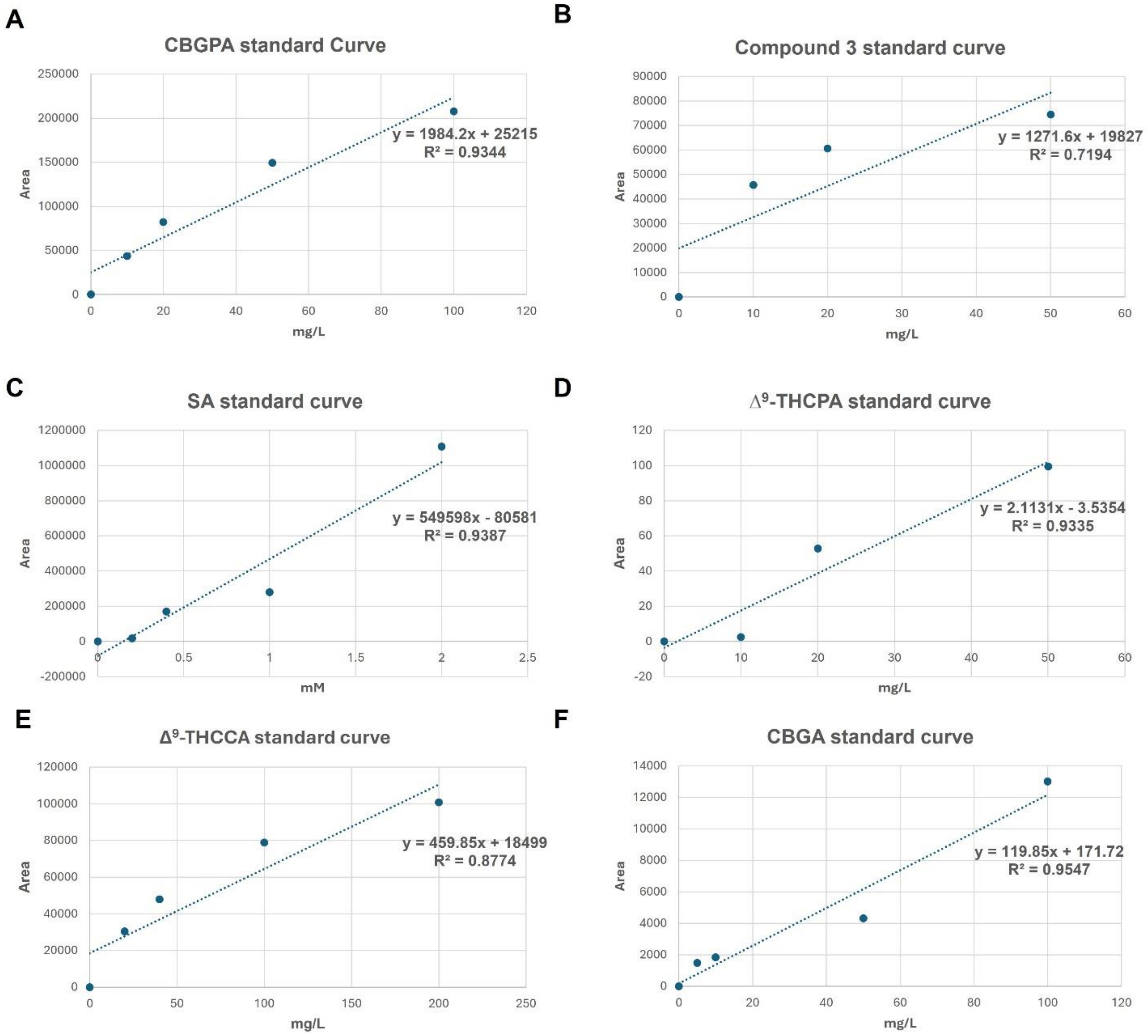

Figure S3. Standard curves of CBGPA, shunt product **3**, SA, Δ^9–THCPA, Δ^9–THCCA, and CBGA. Different concentrations of purified compounds were measured on the HPLC where the area under the peak was recorded and plotted with the concentrations.

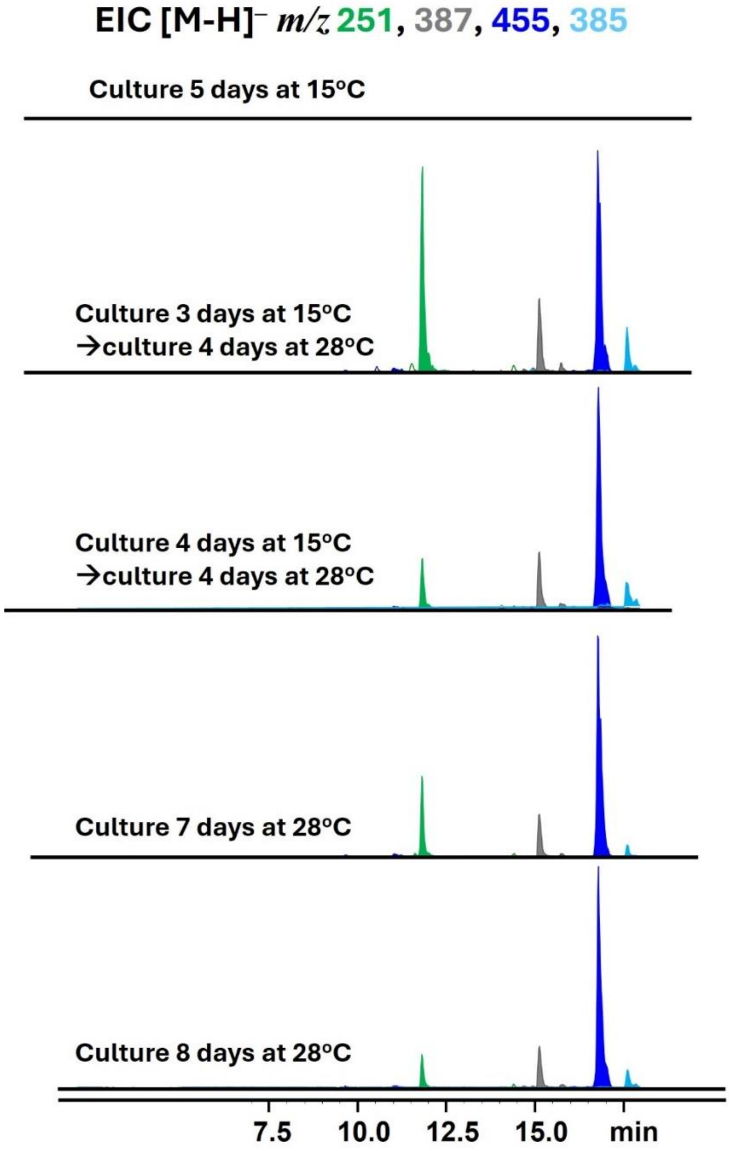

Figure S4. Metabolite analysis of yIO02 transformant at different temperatures. Metabolites profile of yeast transformants expressing Ma_OvaABC + tCSPT4 + NpgA + THCAS. The optimal condition is culturing at 15°C for 3 days and then shifting to 28°C and culture for 4 days. Fermentation at 15 °C did not produce any products.

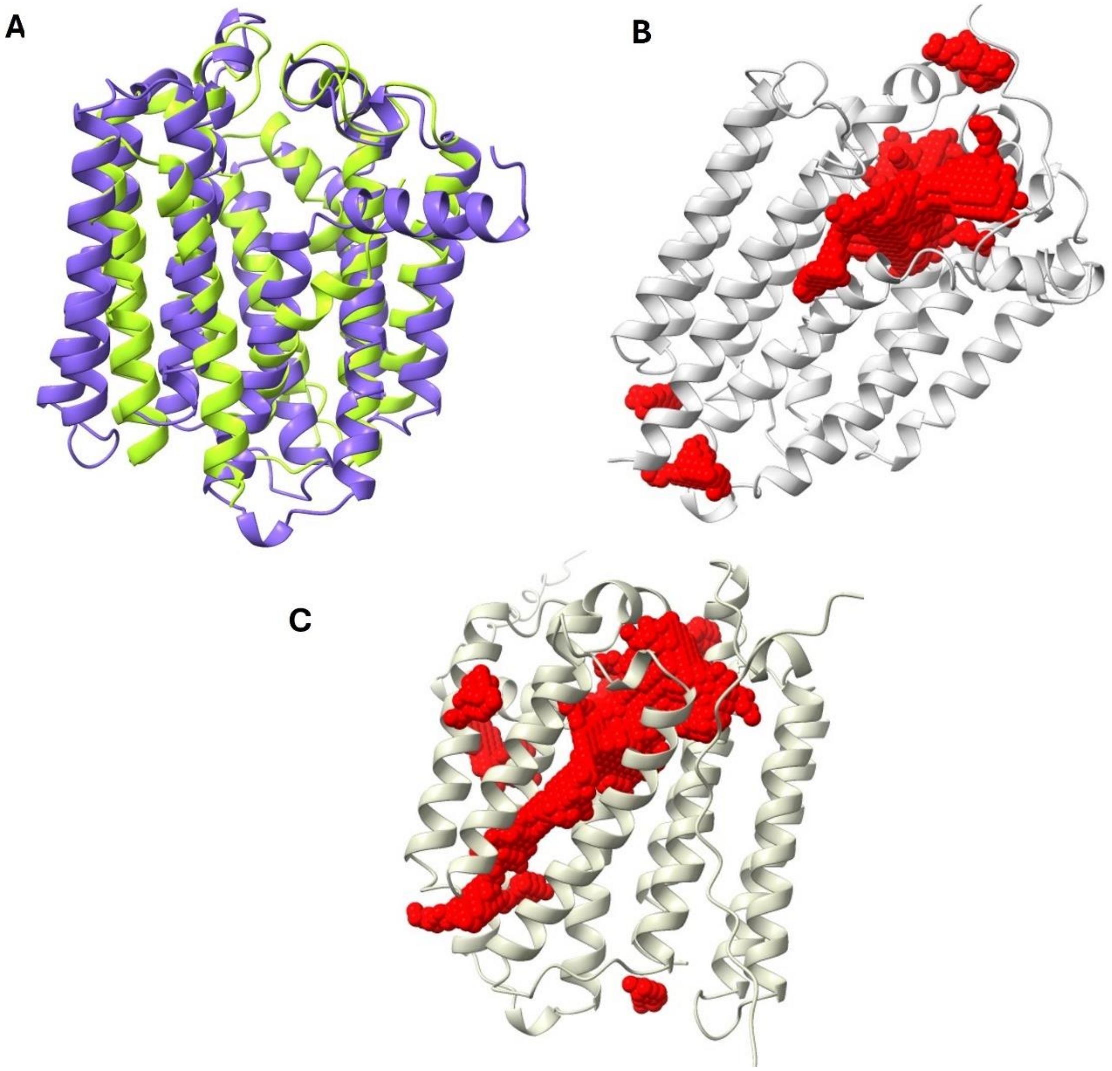

Figure S5. The structural comparison between three UbiA-prenyltransferases, 4OD4, CsPT4 and ColA. A) The structural alignment between crystal structure of 4OD4 (green) and the Alphafold predicted structure of CsPT4 (blue). The two structures align well overall. CsPT4 exhibits bigger central cavity obtaining substrates. B) The predicted binding pocket (red) of ColA. The binding pocket of ColA is smaller which limits the enzyme to accept substrates with long alkyl chains. C) The predicted binding pocket (red) of CsPT4. Similar to 4OD4, the binding pocket of CsPT4 has an unrestricted hydrophobic wall which makes the enzyme non-selective to substrates with various lengths of alkyl chain.[6,7]

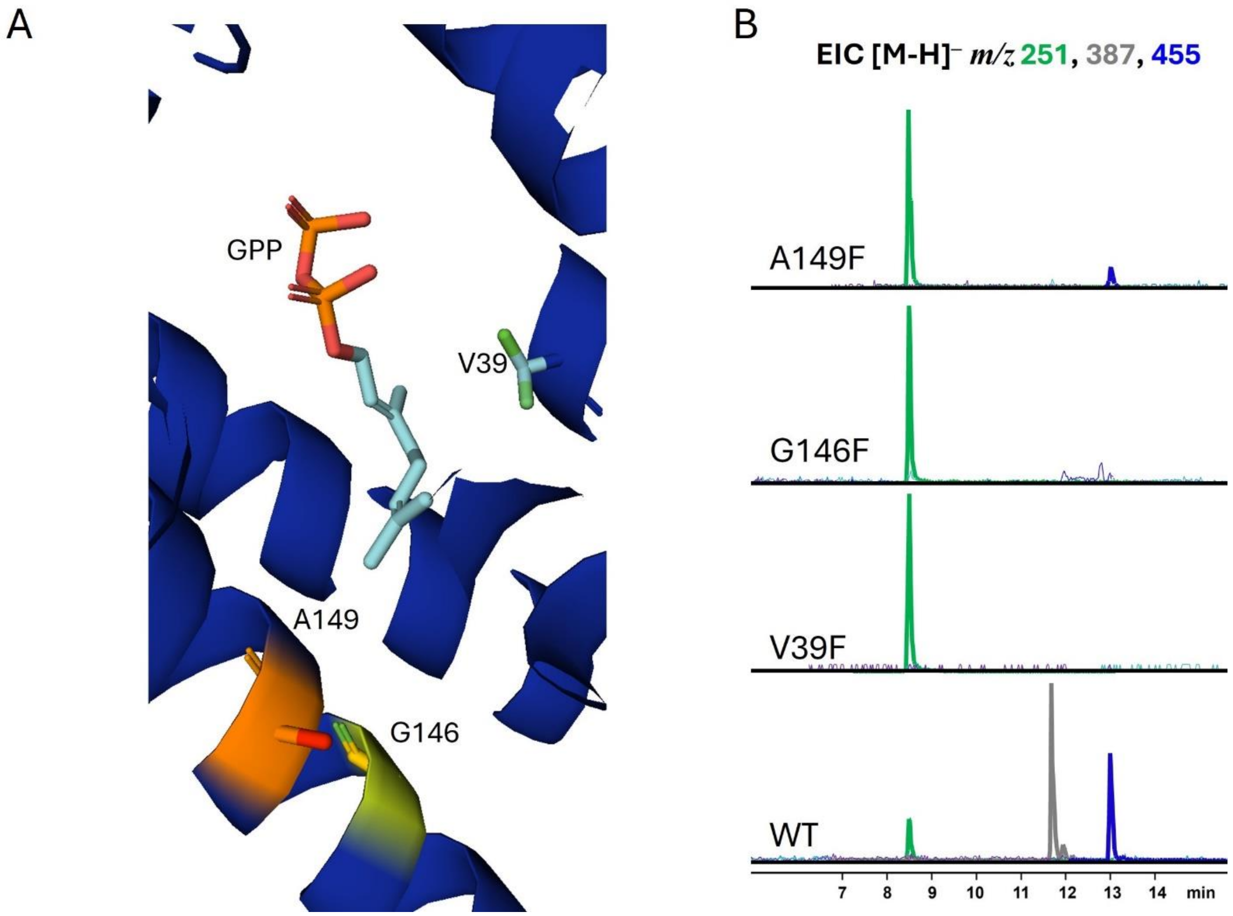

Figure S6. Metabolite analysis of yIO02 transformant expressing tCsPT4 mutatnts. A) Alphafold3 predicted structure of tCsPT4 with GPP in the active site. The spatial orientations of the mutated residues relative to the bound GPP molecule within the active site are highlighted. B) Metabolites profile of yeast transformants co-expressing Ma_OvaABC + mut_tCSPT4 + NpgA + THCAS, and the yeast was cultured under temperature shift conditions. Compared to the WT tCsPT4, the mutants significantly decrease the efficiency or totally abolish the prenylation activities of tCsPT4. Mutations that introduce steric hindrance within the binding pocket impaired the enzyme's ability to accommodate both GPP and FPP, thereby compromising substrate uptake and catalysis.

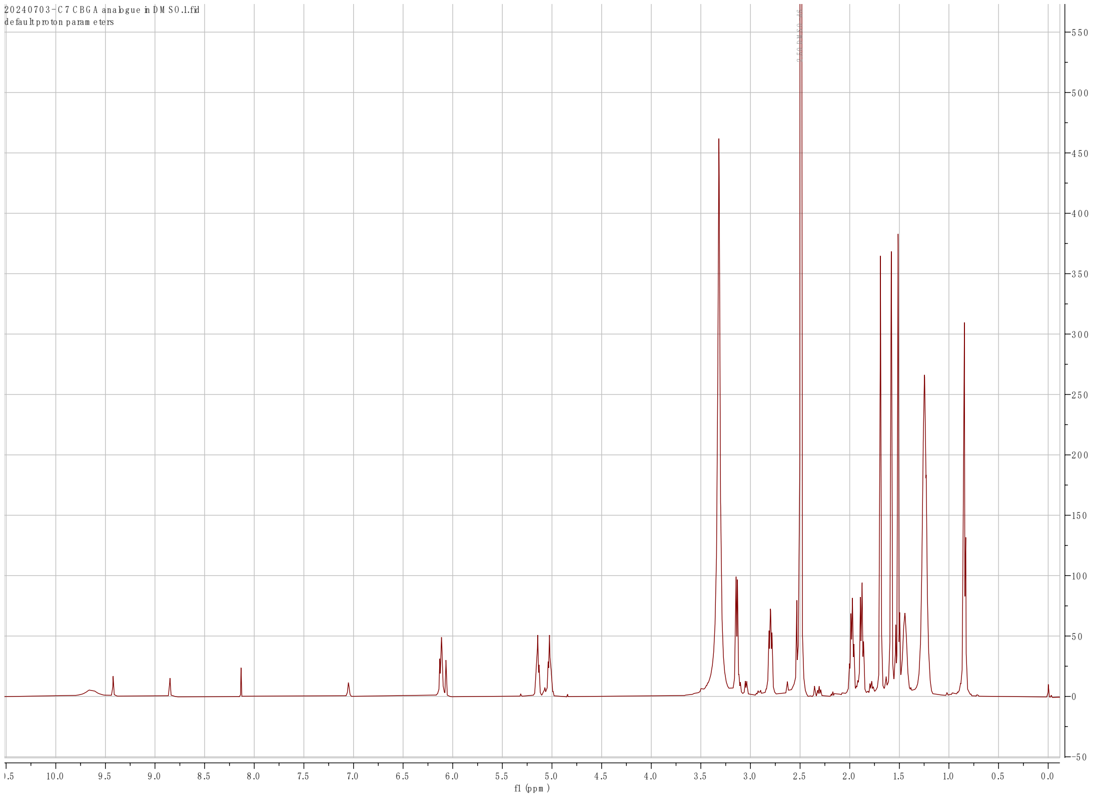

Figure S7 ^{1}H NMR spectrum of compound CBGPA in DMSO-d_6 (500 MHz)

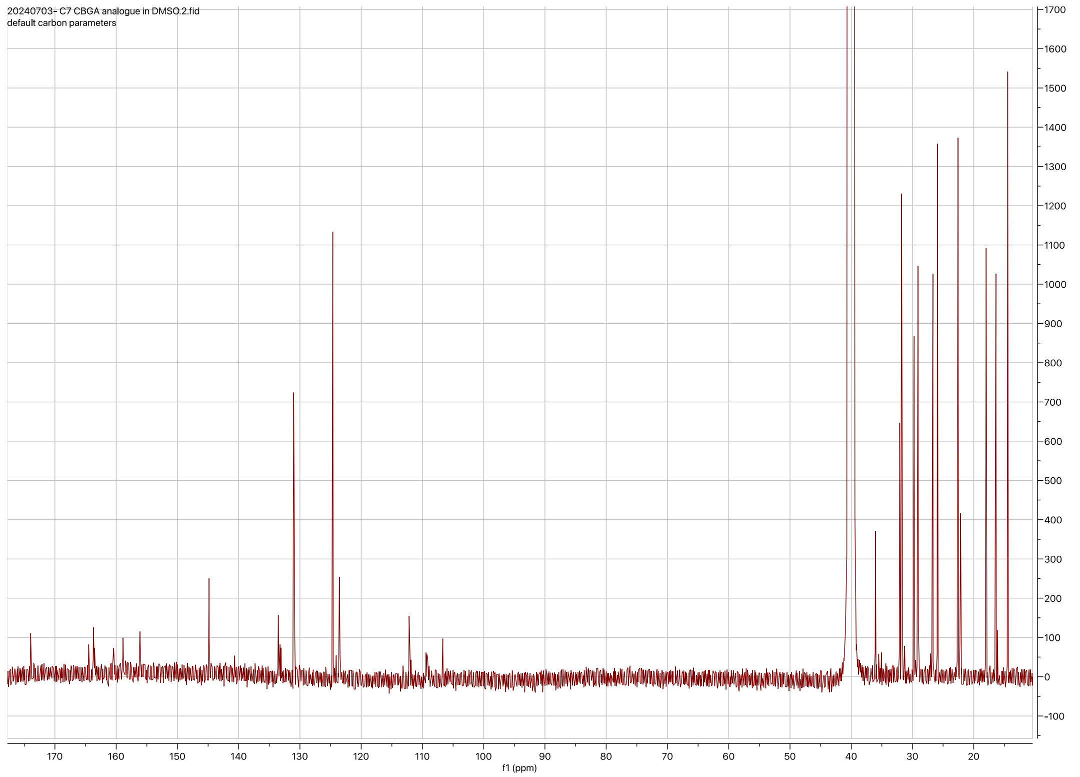

Figure S8 ^{13}C NMR spectrum of compound CBGPA in DMSO-d_6 (125 MHz)

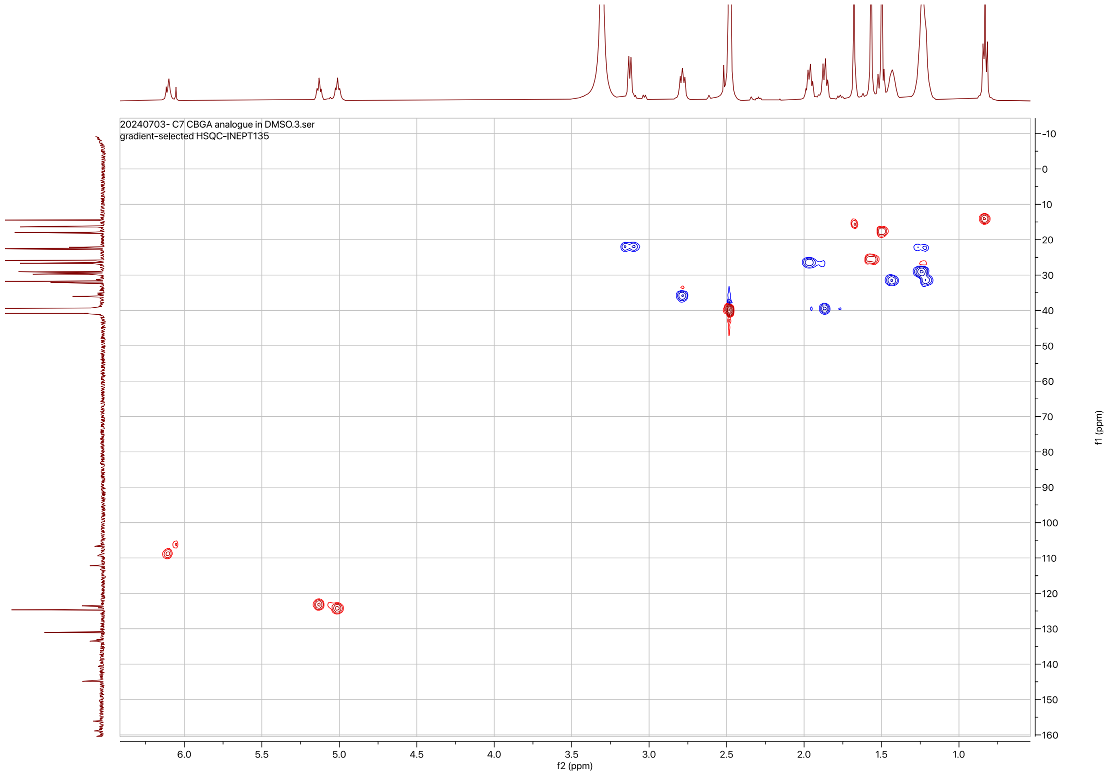

Figure S9 ^{1}H-^{13}C HSQC spectrum of compound CBGPA in DMSO-d_6 (500 MHz)

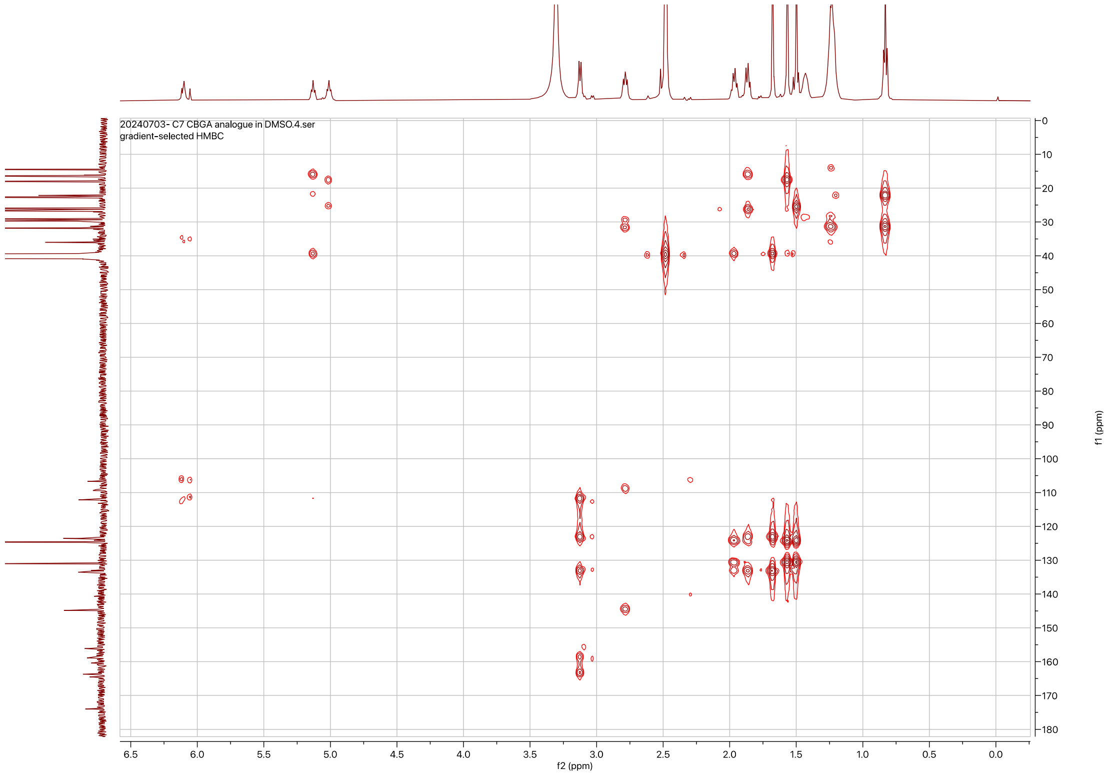

Figure S10 ^{1}H-^{13}C HMBC spectrum of compound CBGPA in DMSO-d_6 (500 MHz)

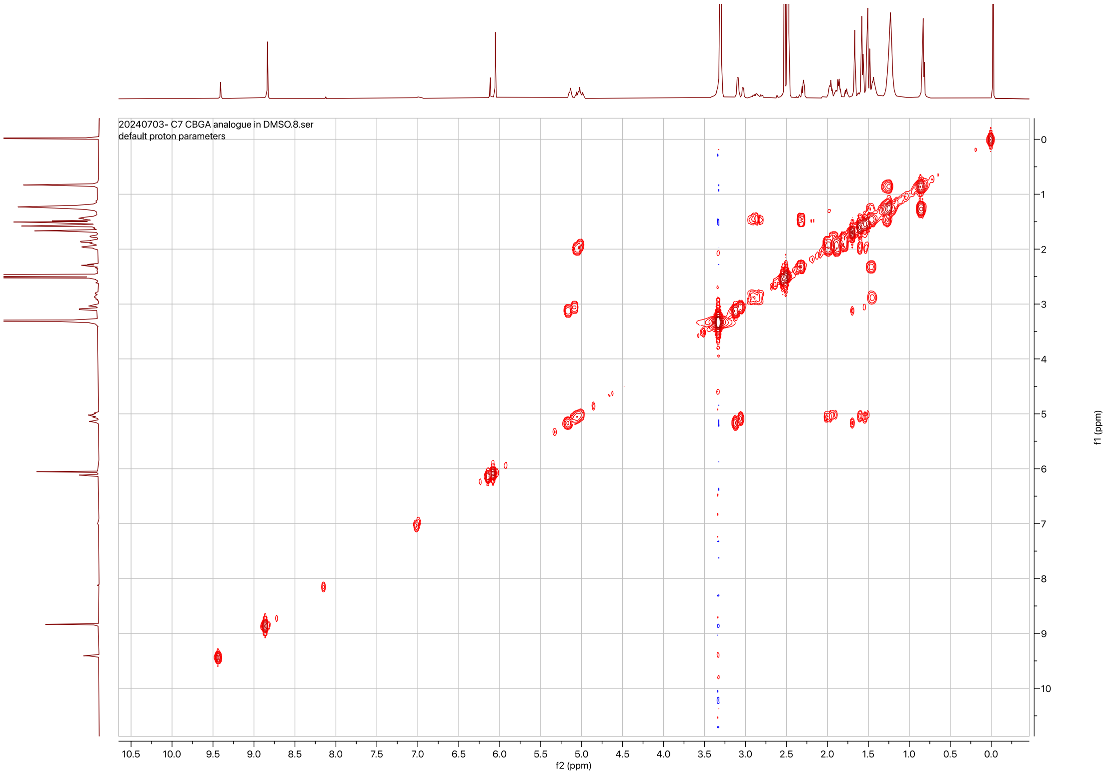

Figure S11 ^{1}H-^{1}H COSY spectrum of compound CBGPA in DMSO- d_6 (500 MHz)

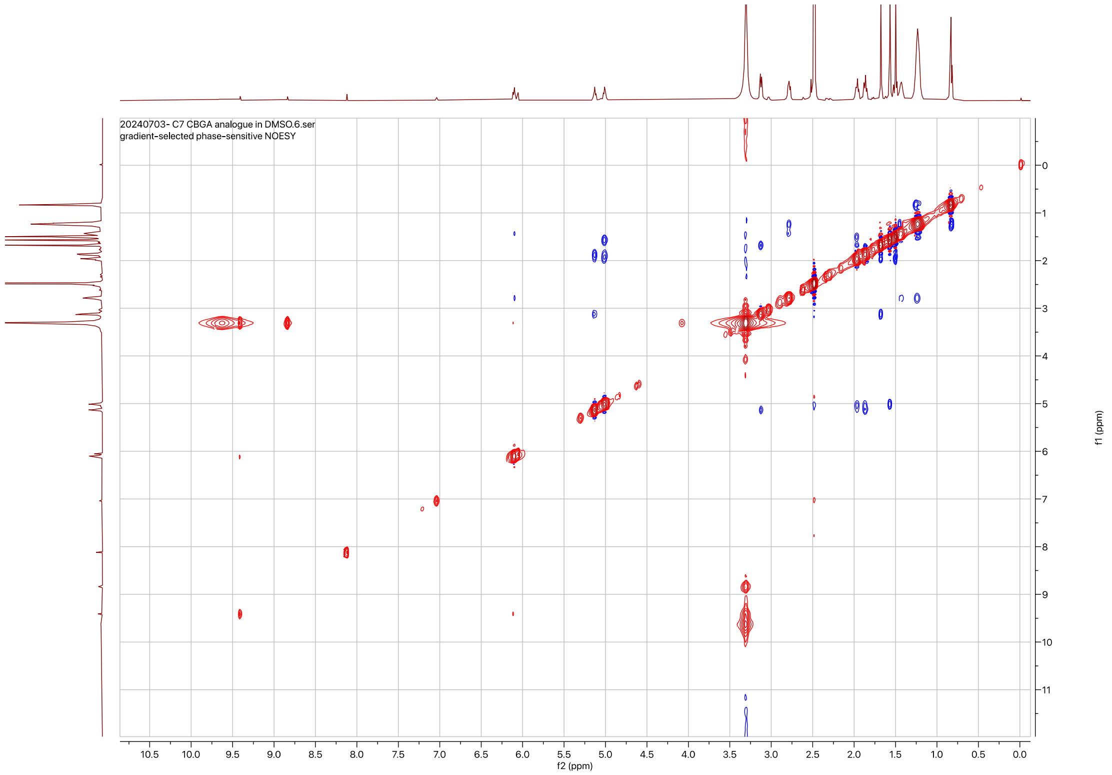

Figure S12 ^{1}H-^{1}H NOESY spectrum of compound CBGPA in DMSO-d_6 (500 MHz)

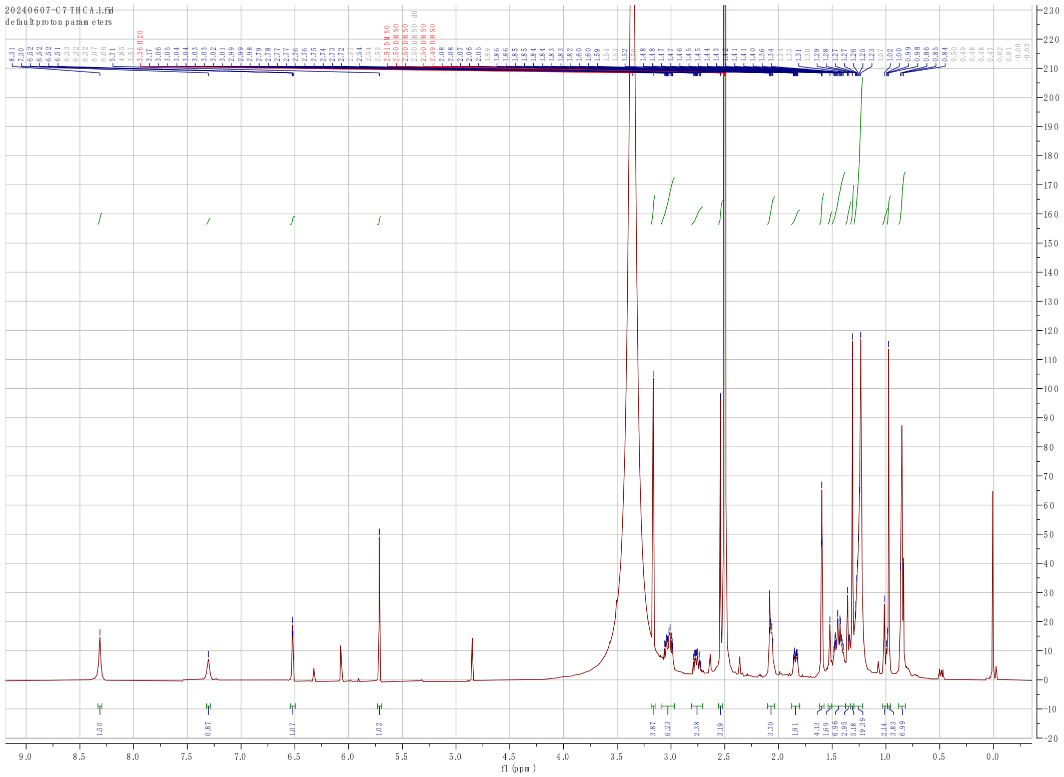

Figure S13 ^{1}H NMR spectrum of compound Δ^9–THCPA in DMSO-d_6 (500 MHz)

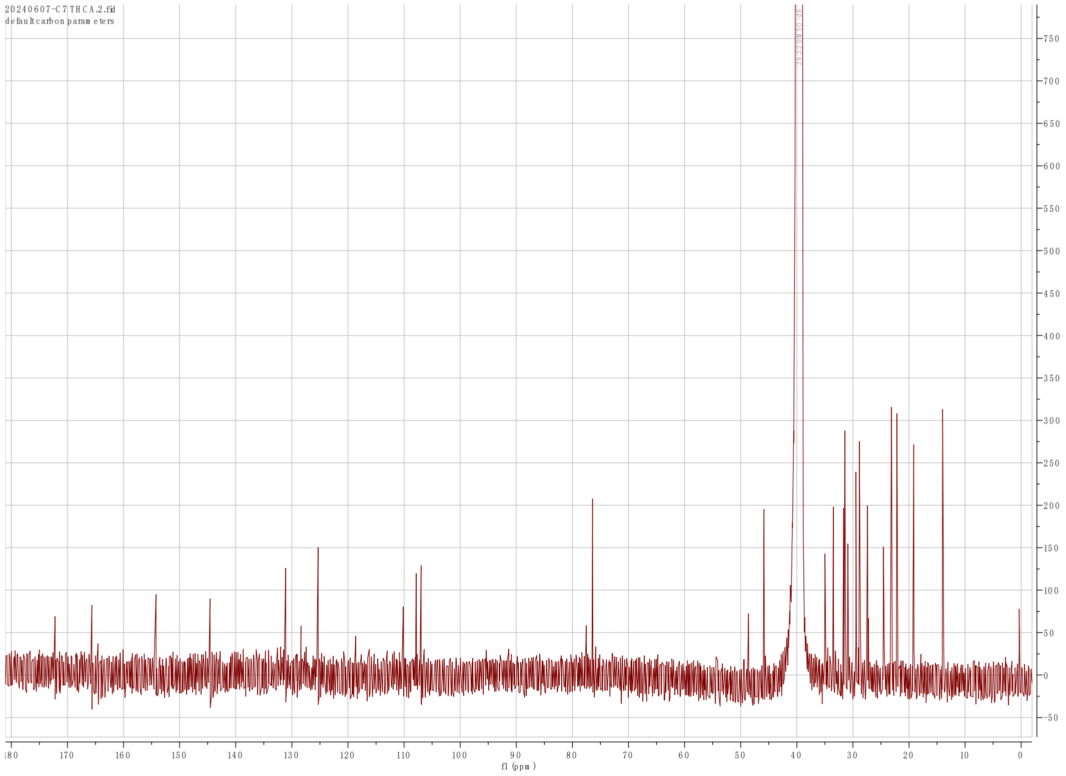

Figure S14 ^{13}C NMR spectrum of compound Δ^9–THCPA in DMSO-d_6 (125 MHz)

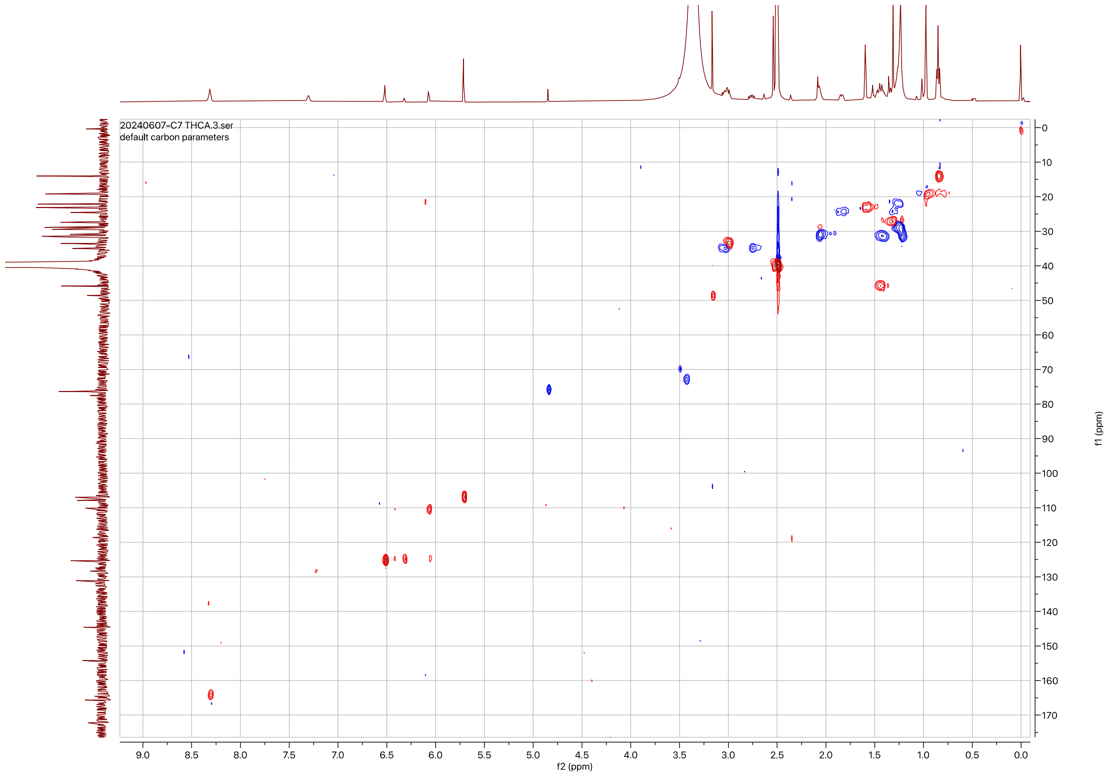

Figure S15 ^{1}H-^{13}C HSQC spectrum of compound Δ^9–THCPA in DMSO-d_6 (500 MHz)

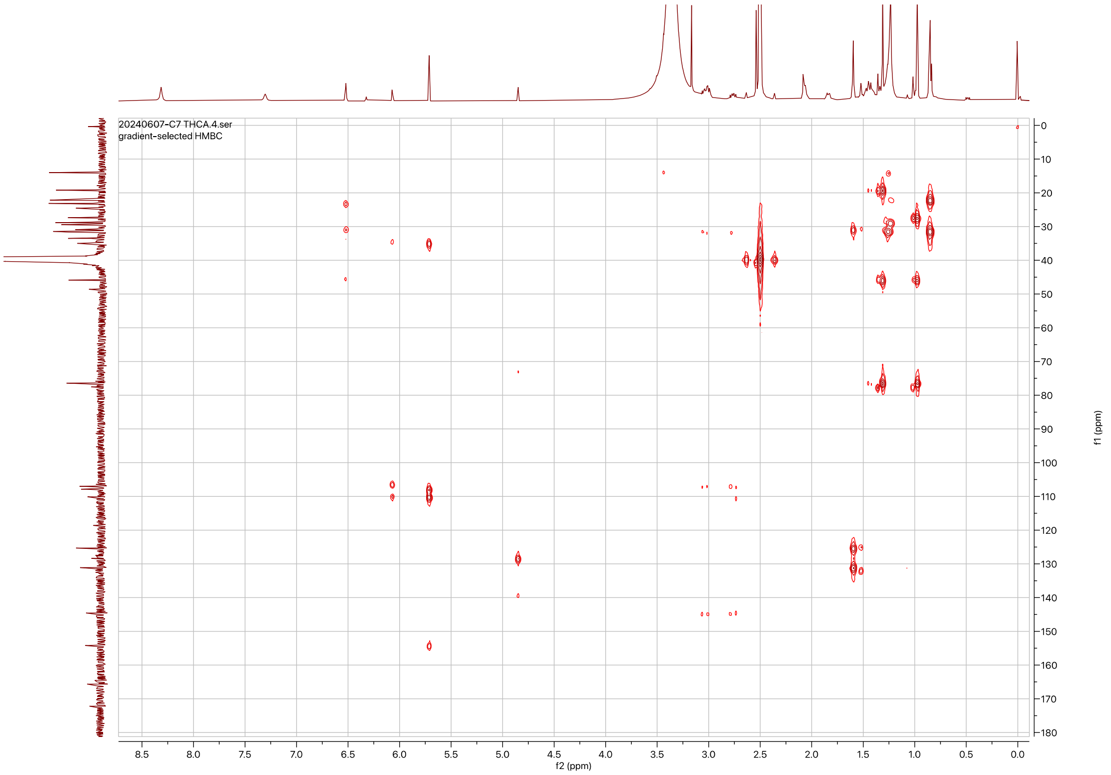

Figure S16 ^{1}H-^{13}C HMBC spectrum of compound Δ[9]–THCPA in DMSO-d_6 (500 MHz)

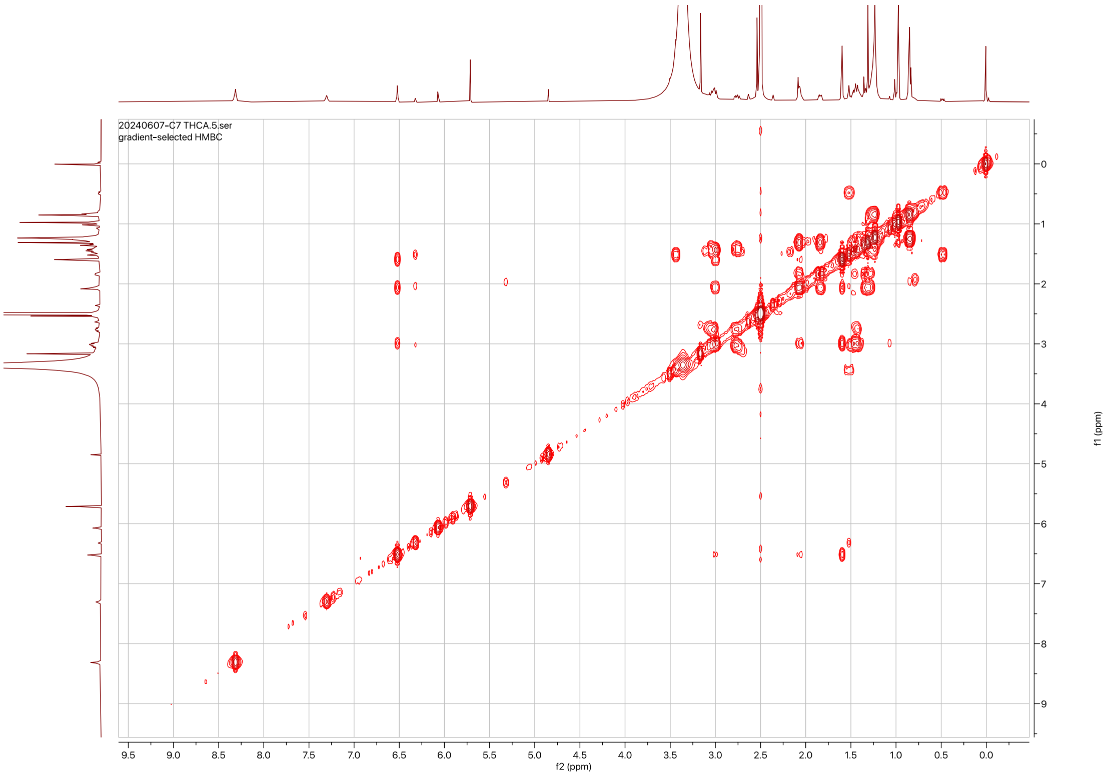

Figure S17 ^{1}H-^{1}H COSY spectrum of compound Δ^{9}–THCPA in DMSO-d_6 (500 MHz)

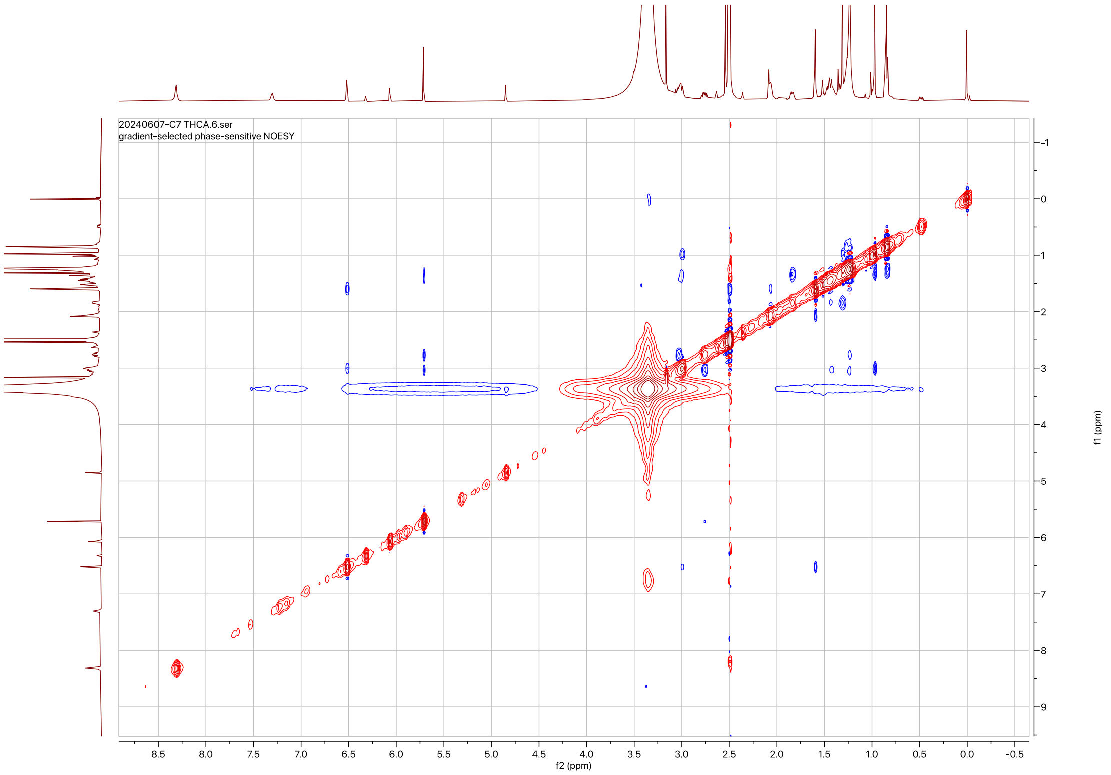

Figure S18 ^{1}H-^{1}H NOESY spectrum of compound Δ^{9}–THCPA in DMSO-d_6 (500 MHz)

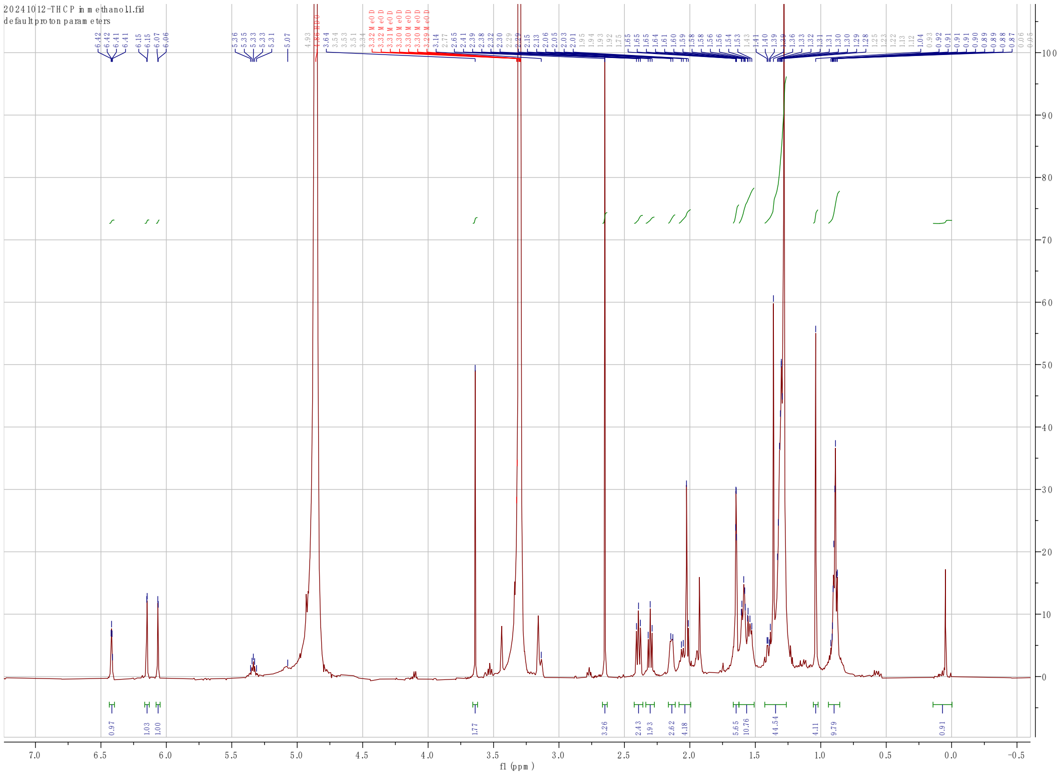

Figure S19 ^1H NMR spectrum of compound Δ^9–THCP in CD_3OD (500 MHz)

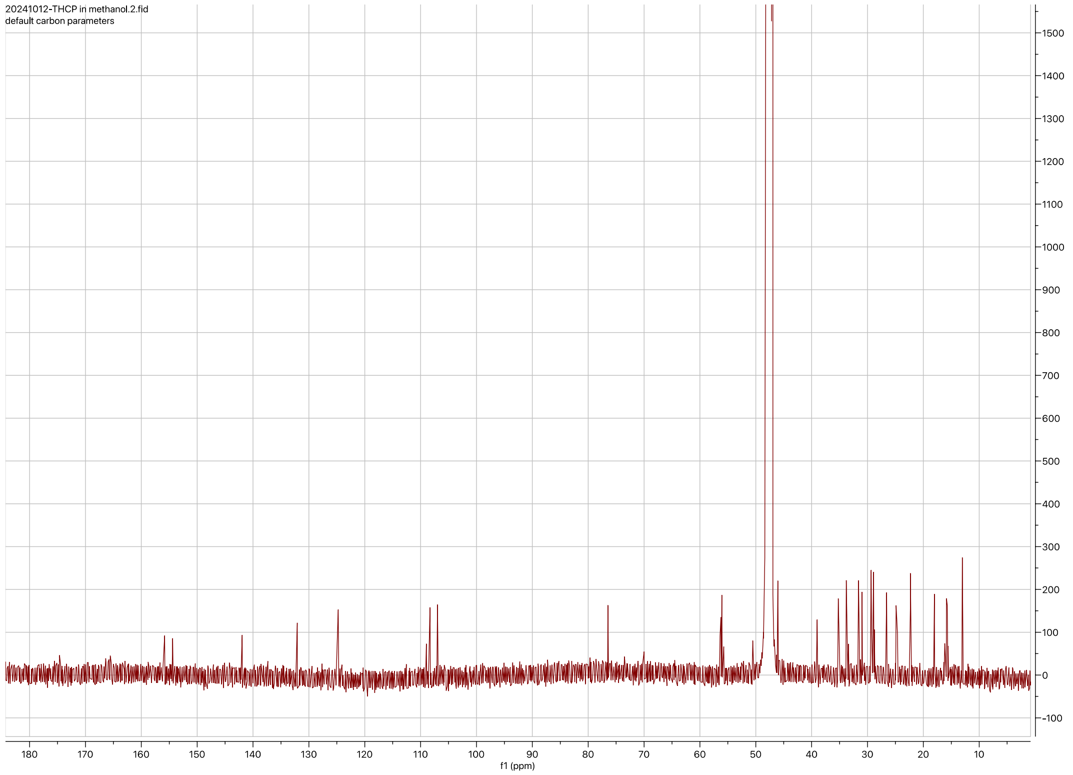

Figure S20 13C NMR spectrum of compound Δ9–THCP in CD3OD (125 MHz)

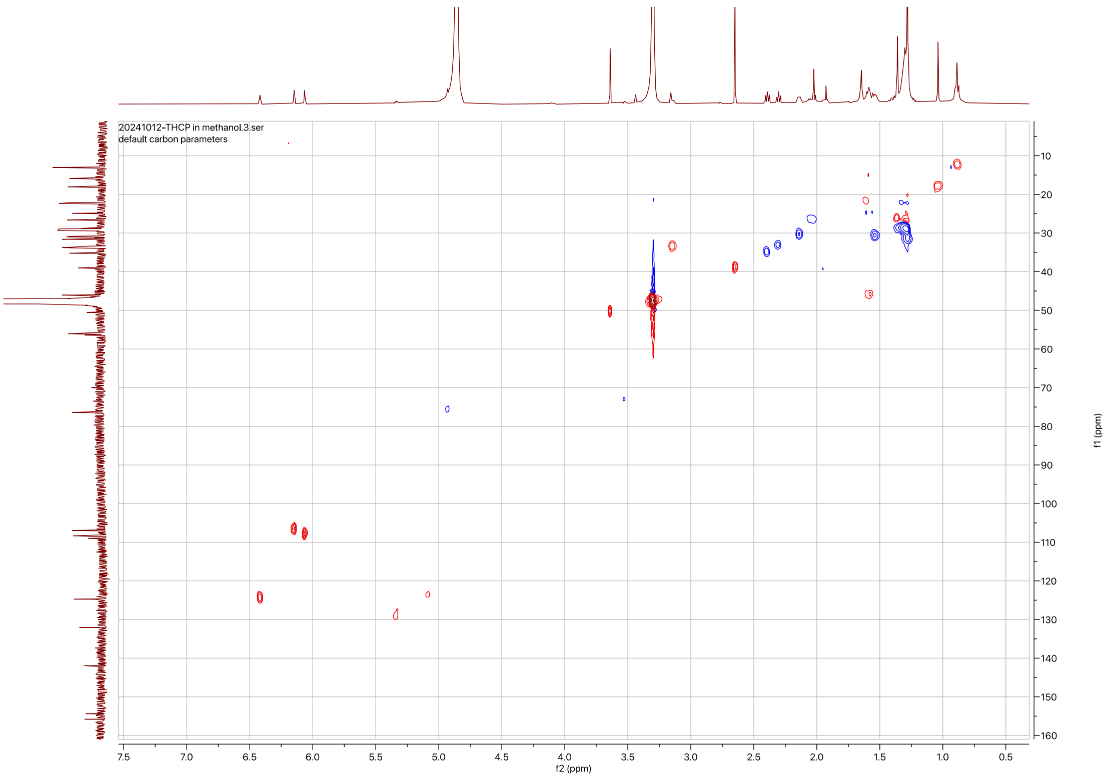

Figure S21 ^{1}H-^{13}C HSQC spectrum of compound Δ^9–THCP in CD_3OD (500 MHz)

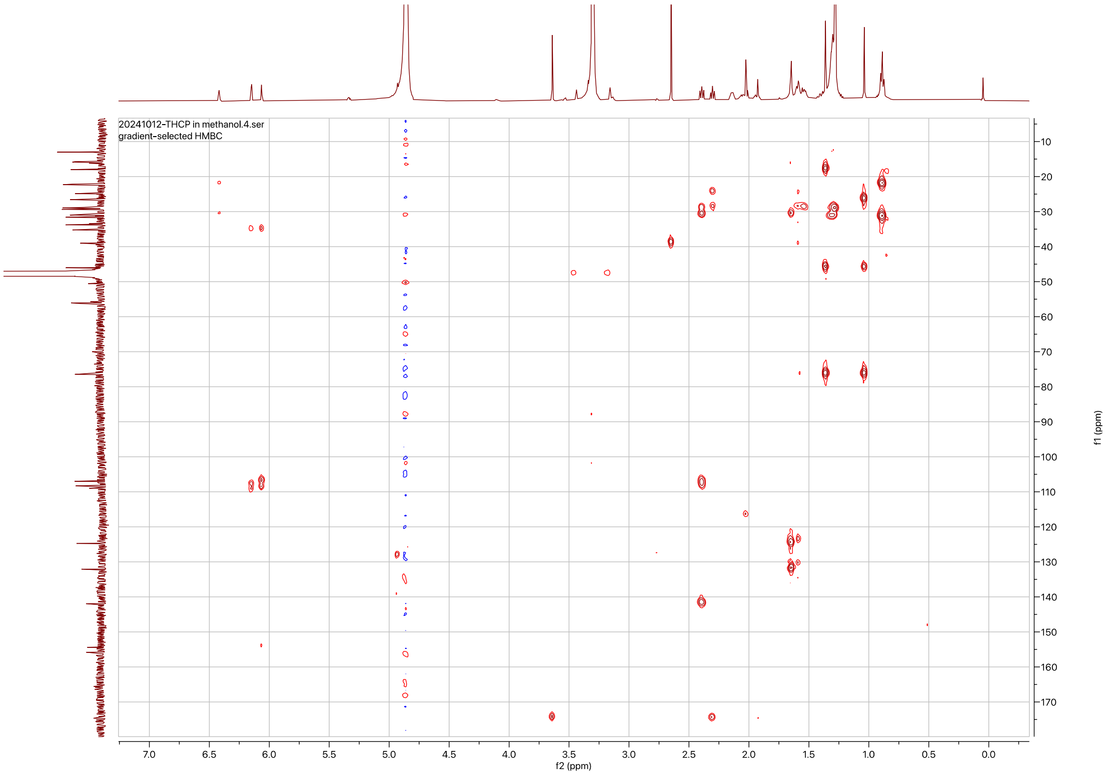

Figure S22 ^{1}H-^{13}C HMBC spectrum of compound Δ^{9}–THCP in CD_3OD (500 MHz)

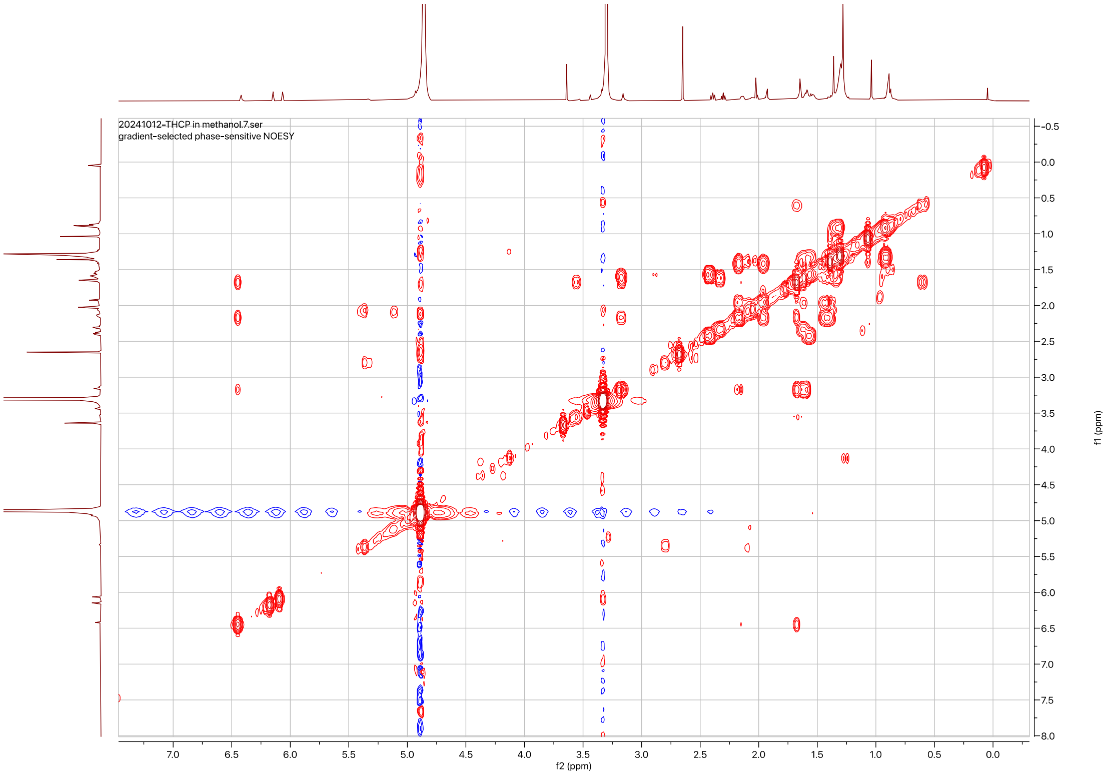

Figure S23 ^{1}H-^{1}H COSY spectrum of compound Δ^9–THCP in CD_3OD (500 MHz)

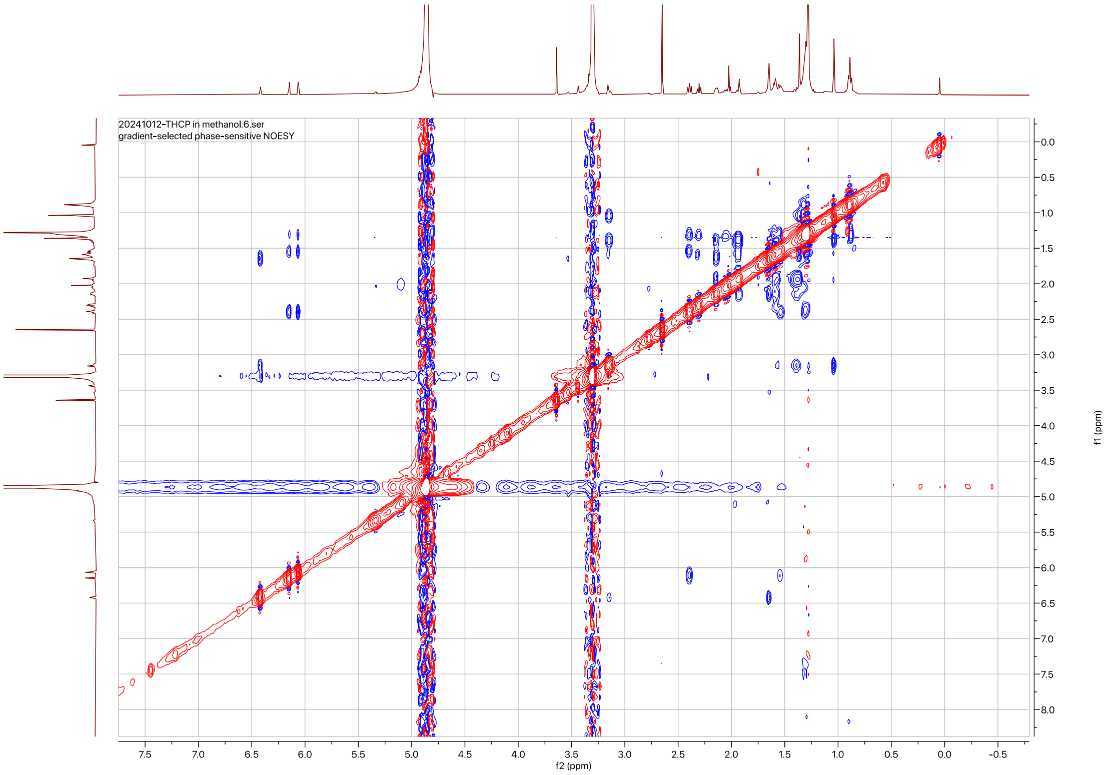

Figure S24 ^1H-^1H NOESY spectrum of compound Δ^9–THCP in CD_3OD (500 MHz)

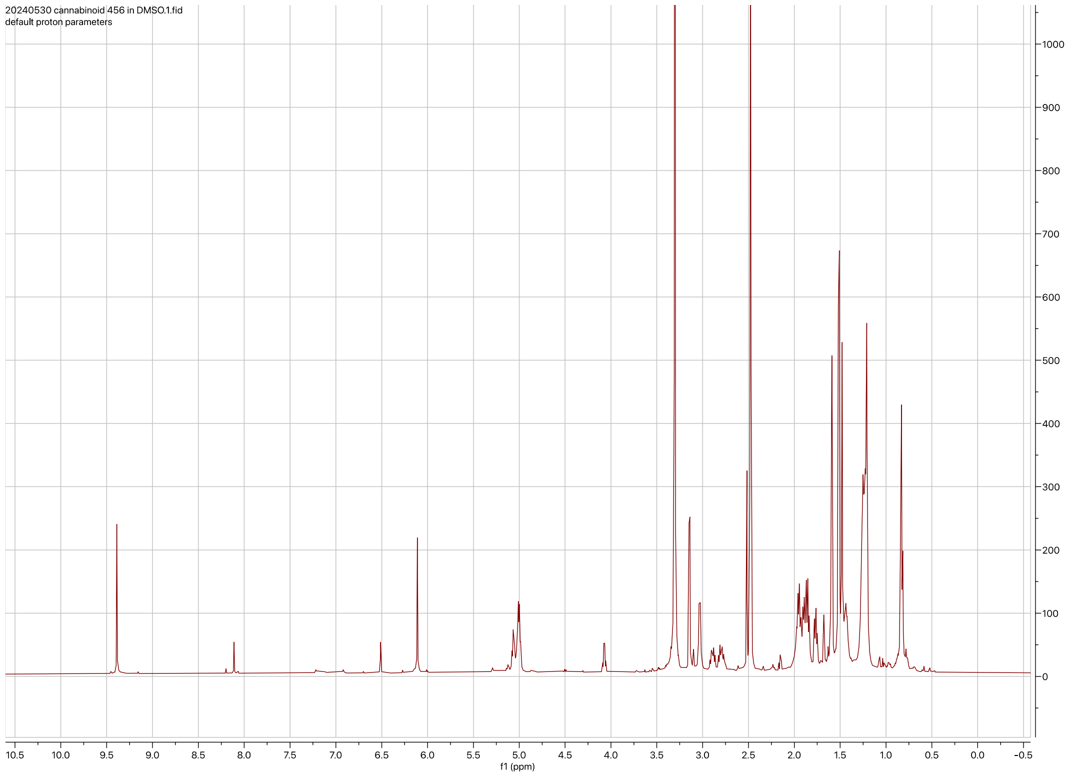

Figure S25 ^{1}H NMR spectrum of compound **3** in DMSO-d_6 (500 MHz)

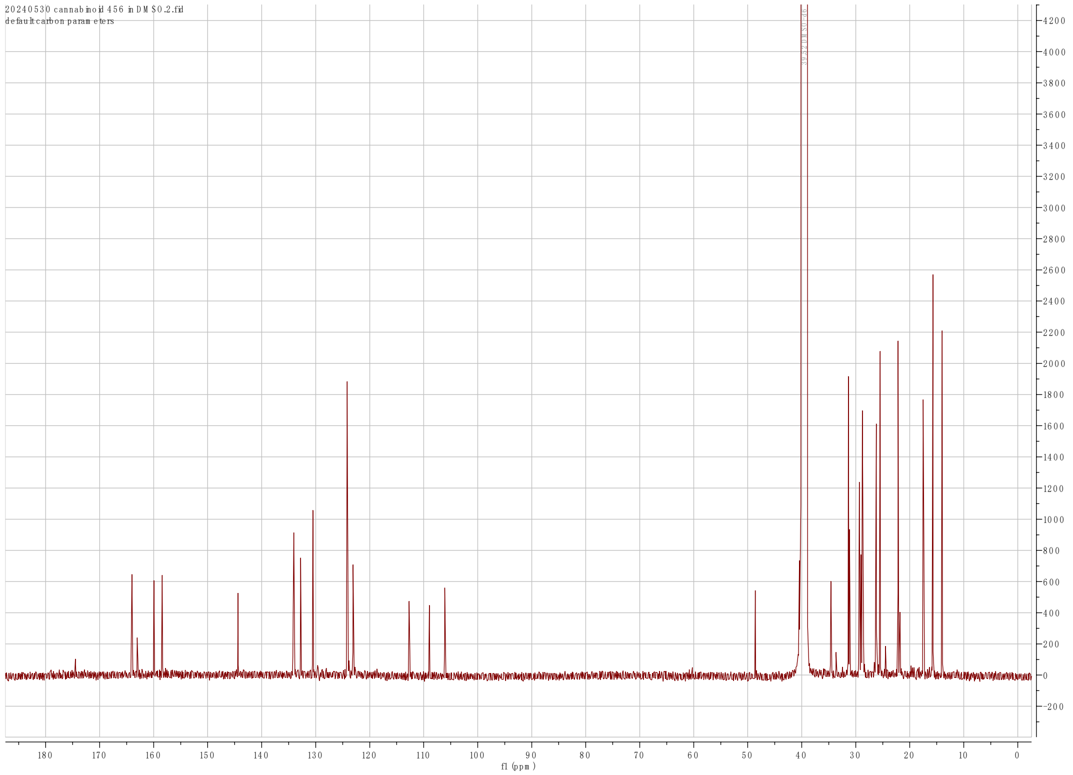

Figure S26 ^{13}C NMR spectrum of compound **3** in DMSO-d_6 (125 MHz)

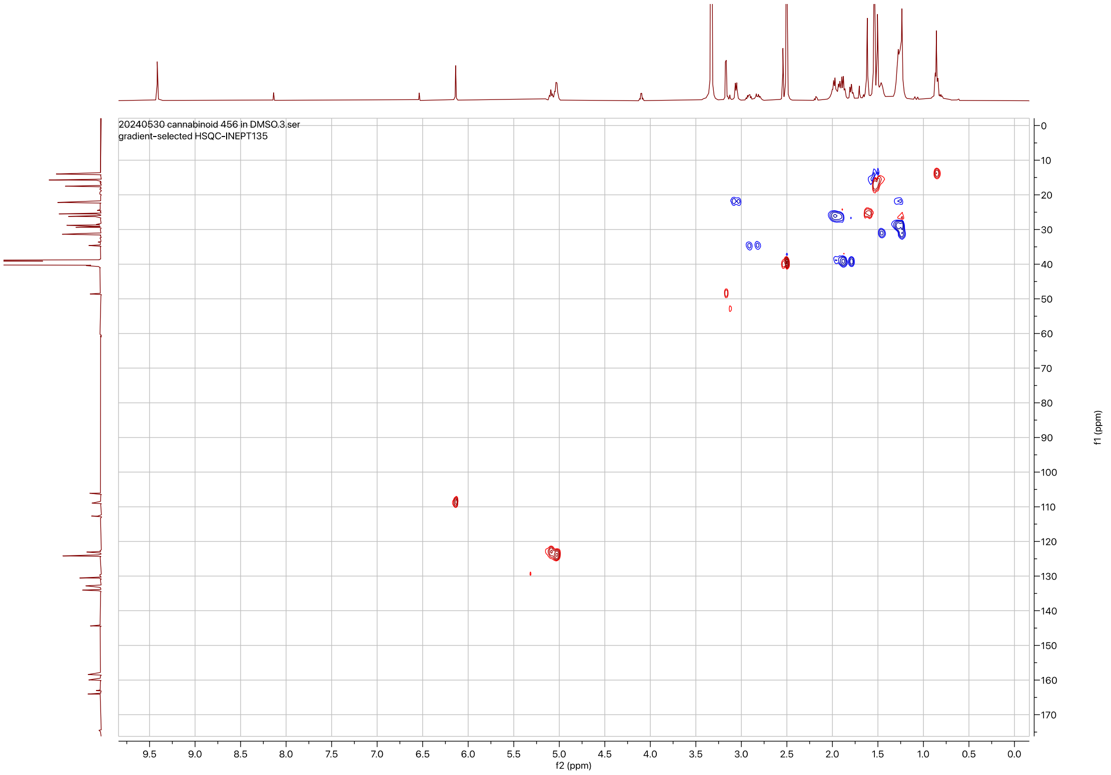

Figure S27 ^{1}H-^{13}C HSQC spectrum of compound **3** in DMSO-d_6 (500 MHz)

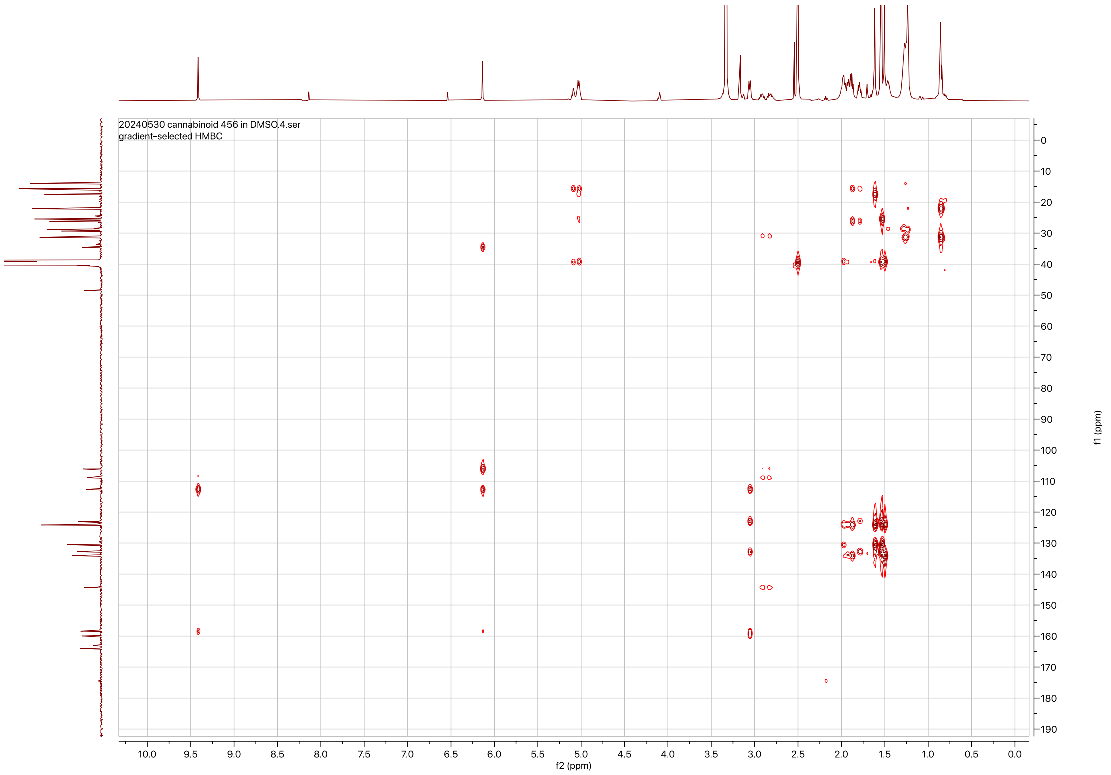

Figure S28 ^{1}H-^{13}C HMBC spectrum of compound **3** in DMSO-d_6 (500 MHz)

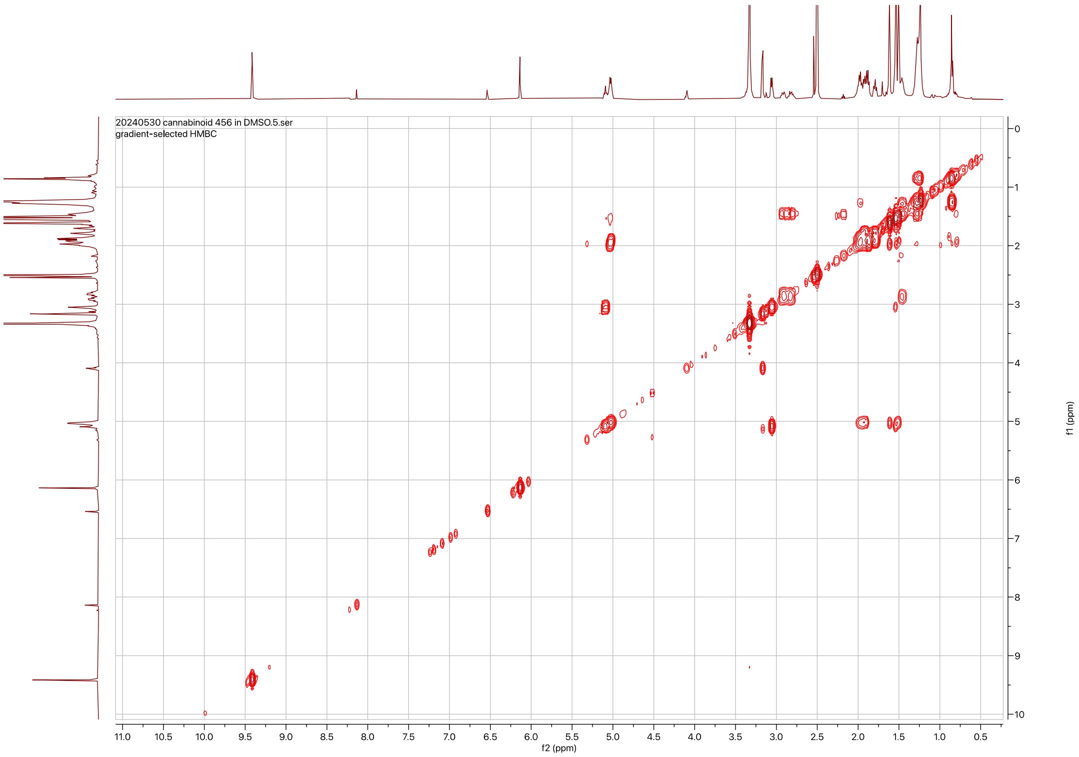

Figure S29 ^{1}H-^{1}H COSY spectrum of compound **3** in DMSO-d_6 (500 MHz)

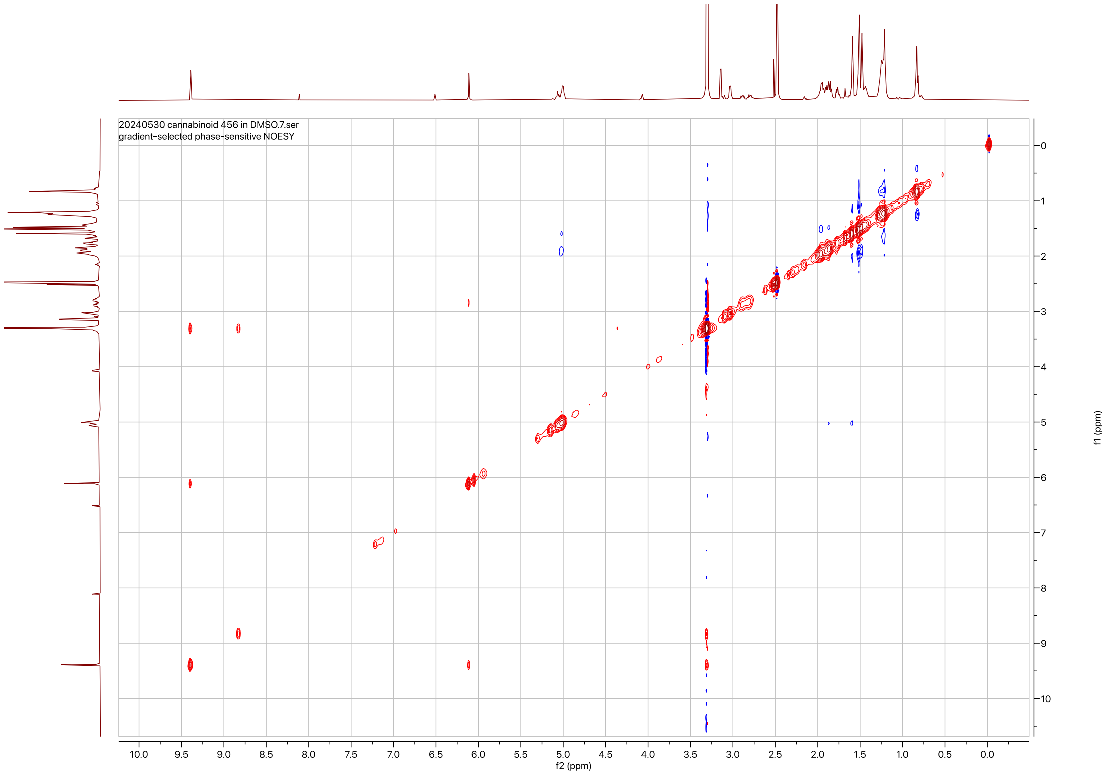

Figure S30 1H-1H NOESY spectrum of compound **3** in DMSO- d_6 (500 MHz)

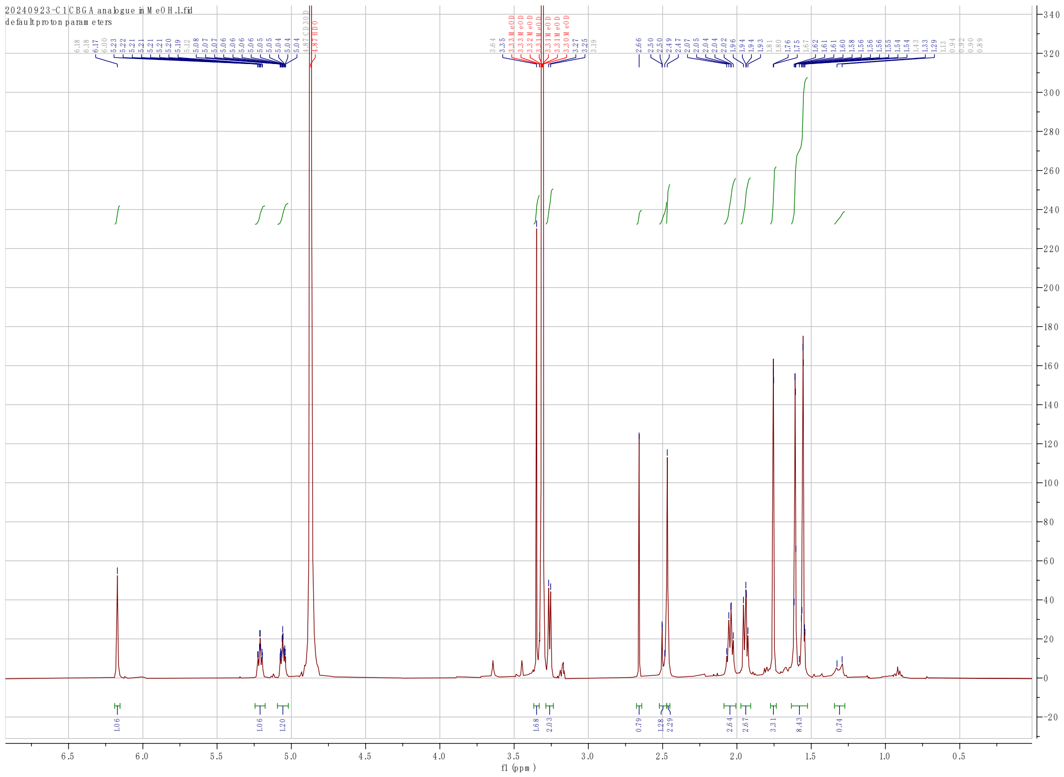

Figure S31 ^{1}H NMR spectrum of compound CBGCA in CD_3OD (500 MHz)

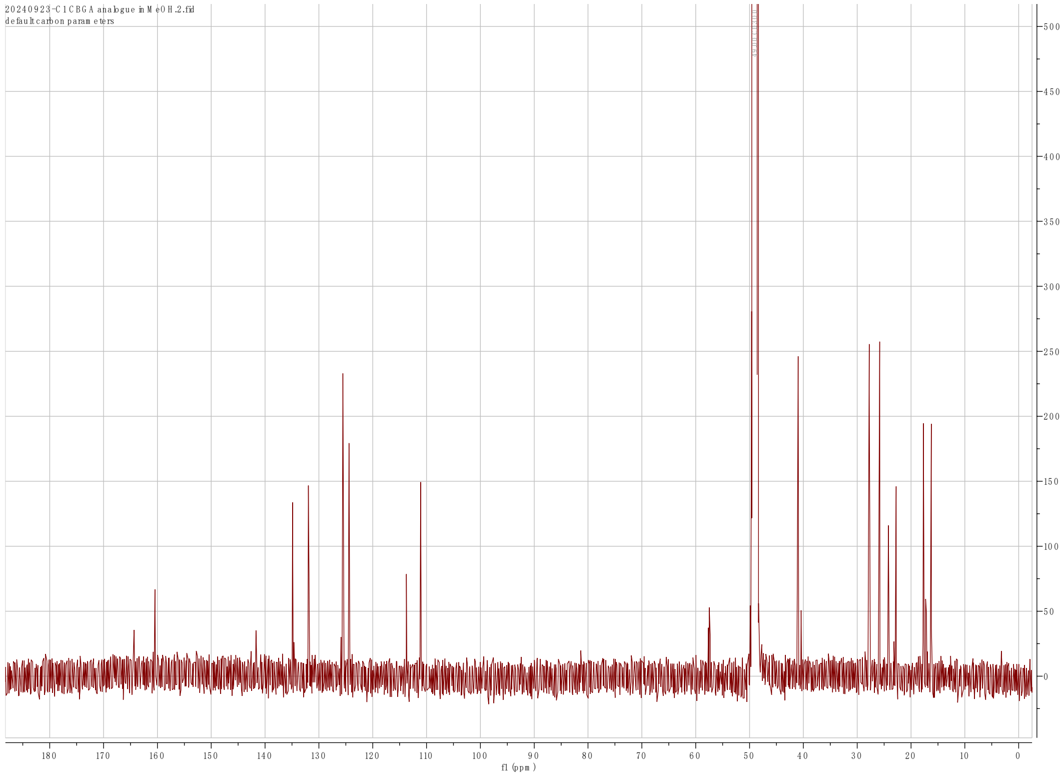

Figure S32 ^{13}C NMR spectrum of compound CBGCA in CD_3OD (125 MHz)

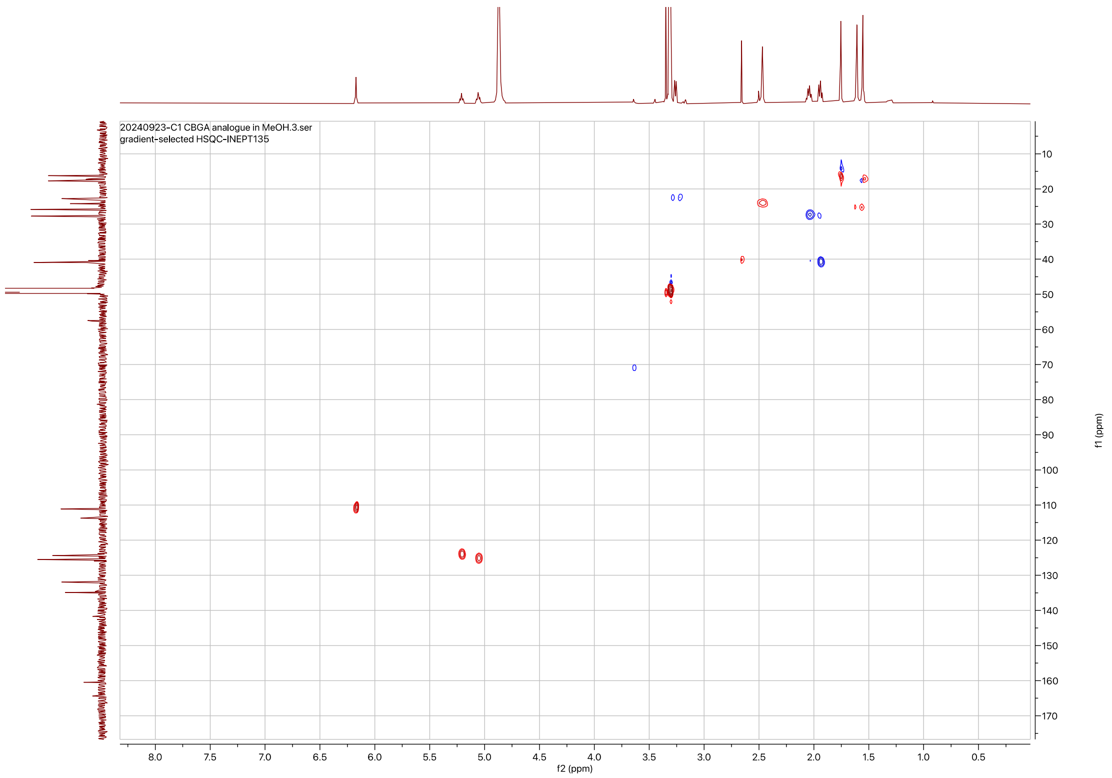

Figure S33 ^{1}H-^{13}C HSQC spectrum of compound CBGCA in CD_3OD (500 MHz)

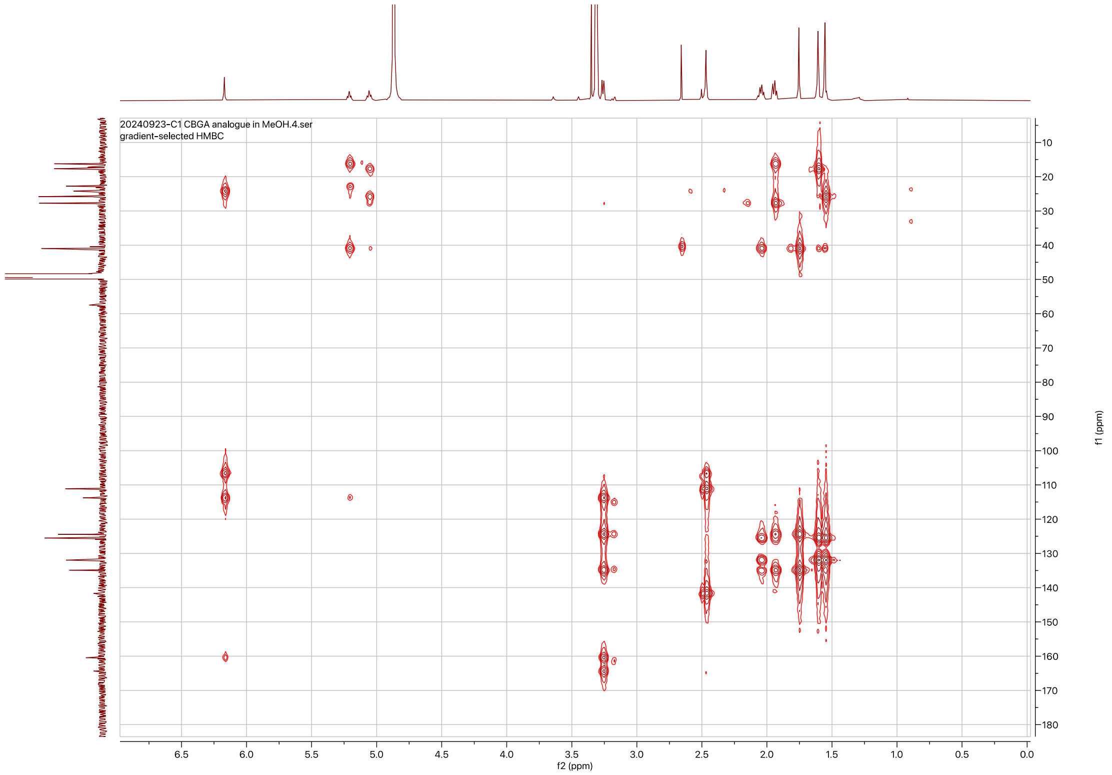

Figure S34 ^{1}H-^{13}C HMBC spectrum of compound CBGCA in CD_3OD (500 MHz)

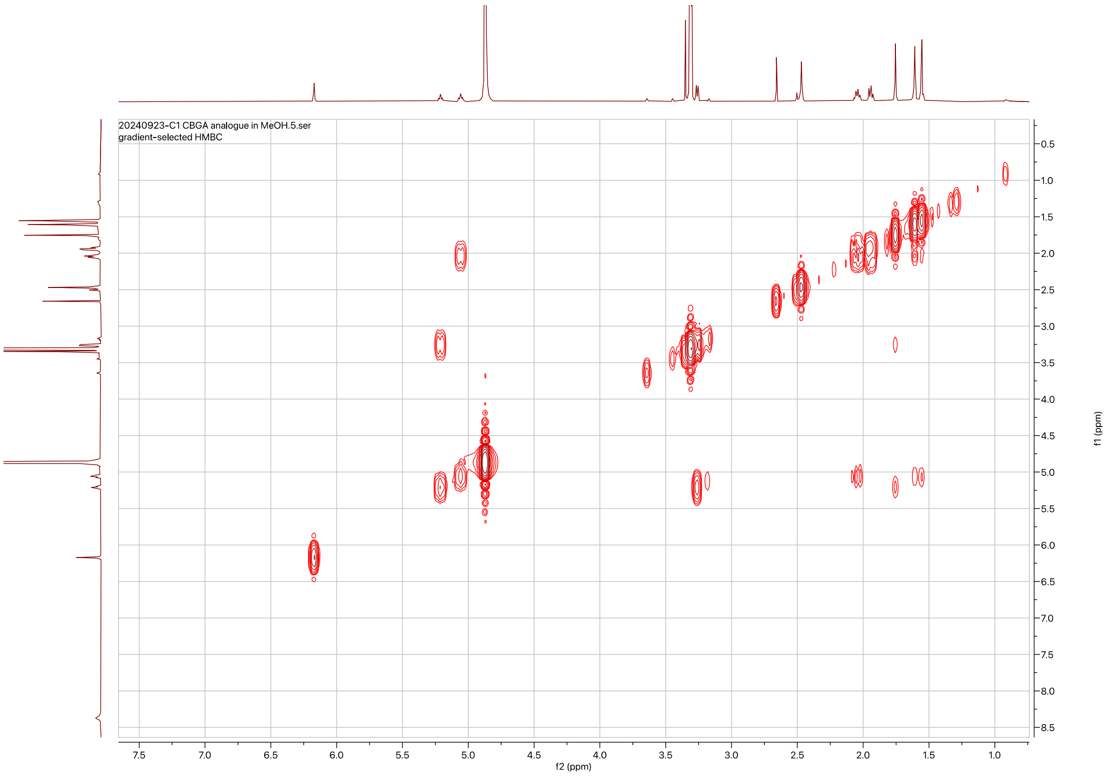

Figure S35 ^1H-^1H COSY spectrum of compound CBGCA in CD_3OD (500 MHz)

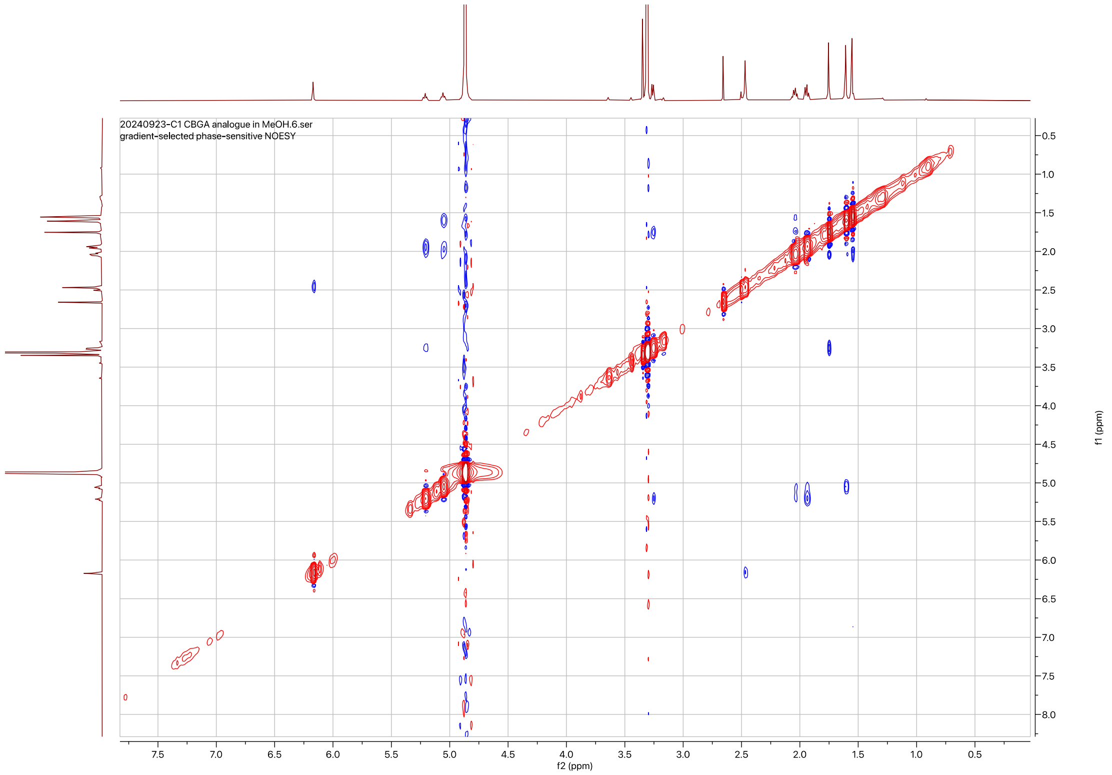

Figure S36 ¹H-¹H NOESY spectrum of compound CBGCA in CD_3OD (500 MHz)

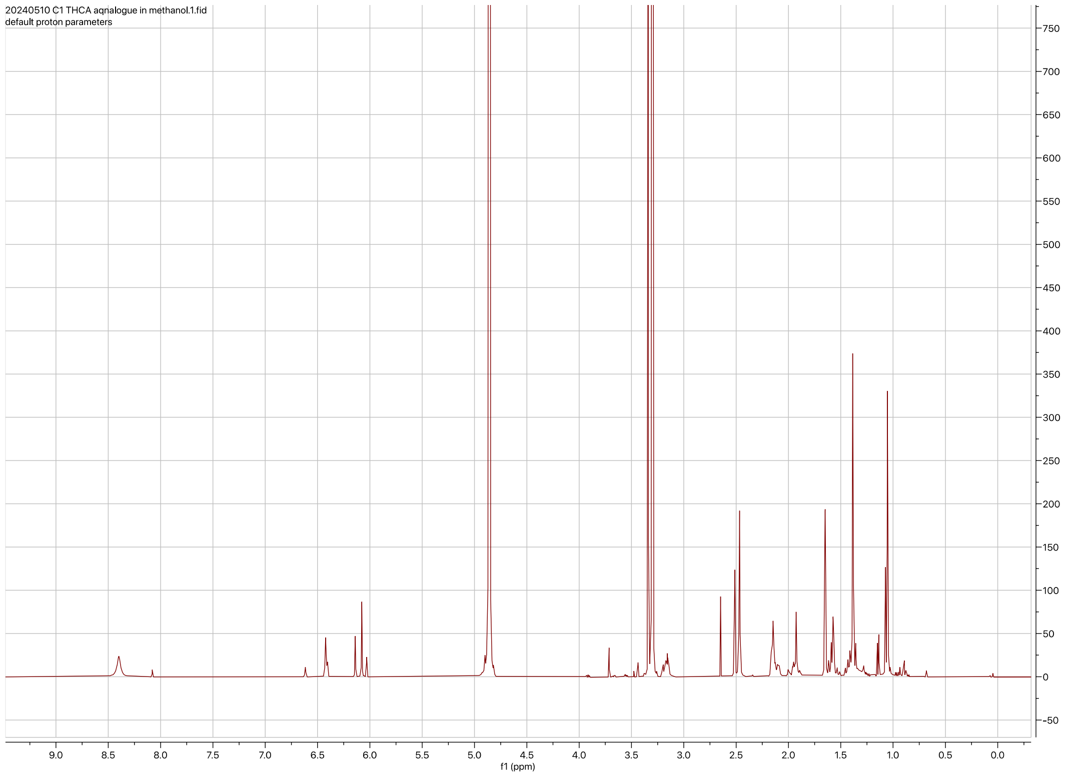

Figure S37 ^{1}H NMR spectrum of compound Δ^{9}–THCCA in CD_3OD (500 MHz)

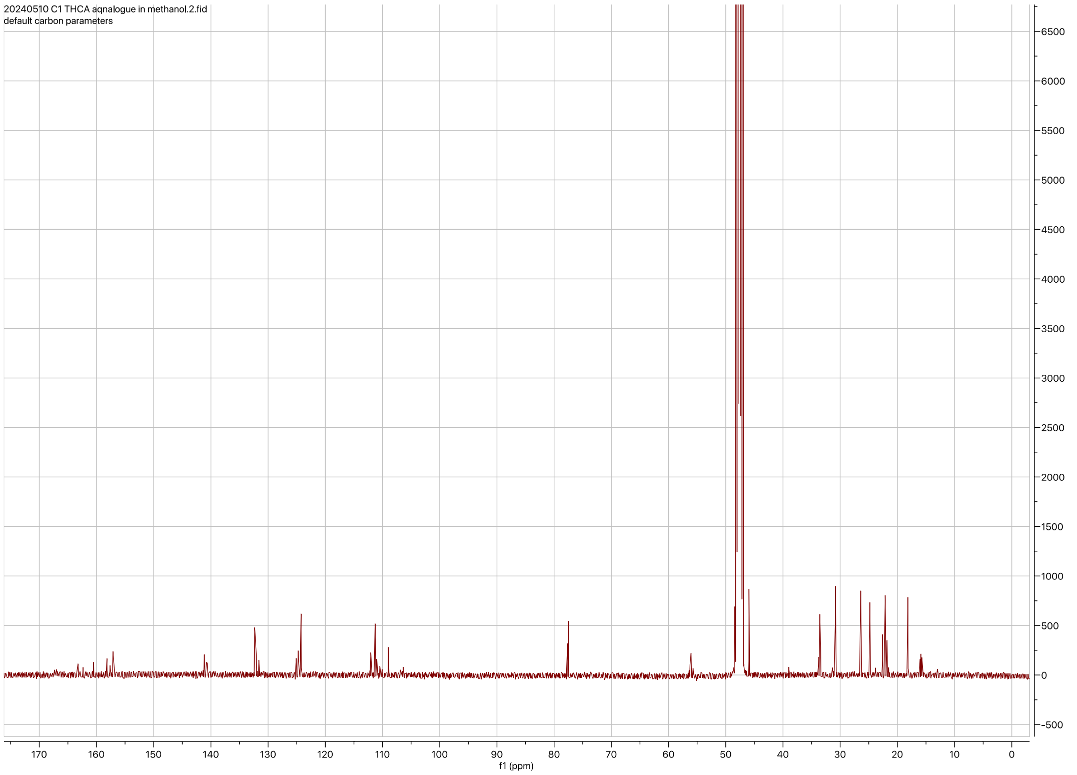

Figure S38 ^{13}C NMR spectrum of compound Δ^9–THCCA in CD_3OD (125 MHz)

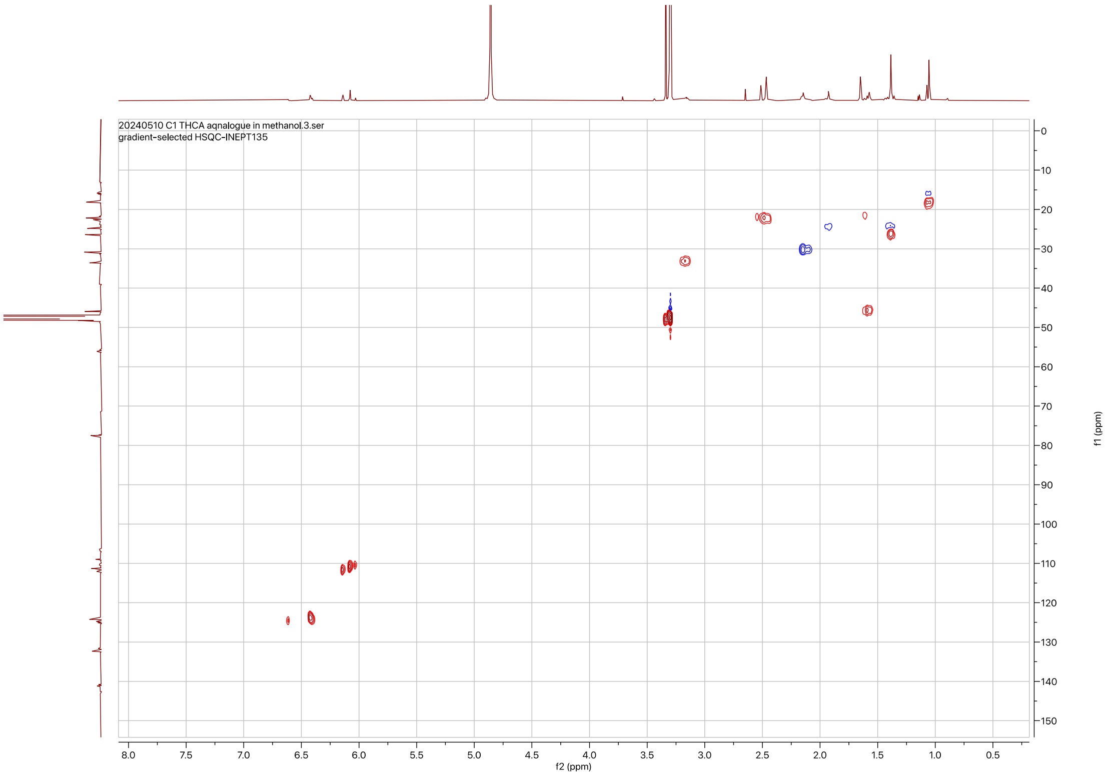

Figure S39 ^{1}H-^{13}C HSQC spectrum of compound Δ[9]–THCCA in CD_3OD (500 MHz)

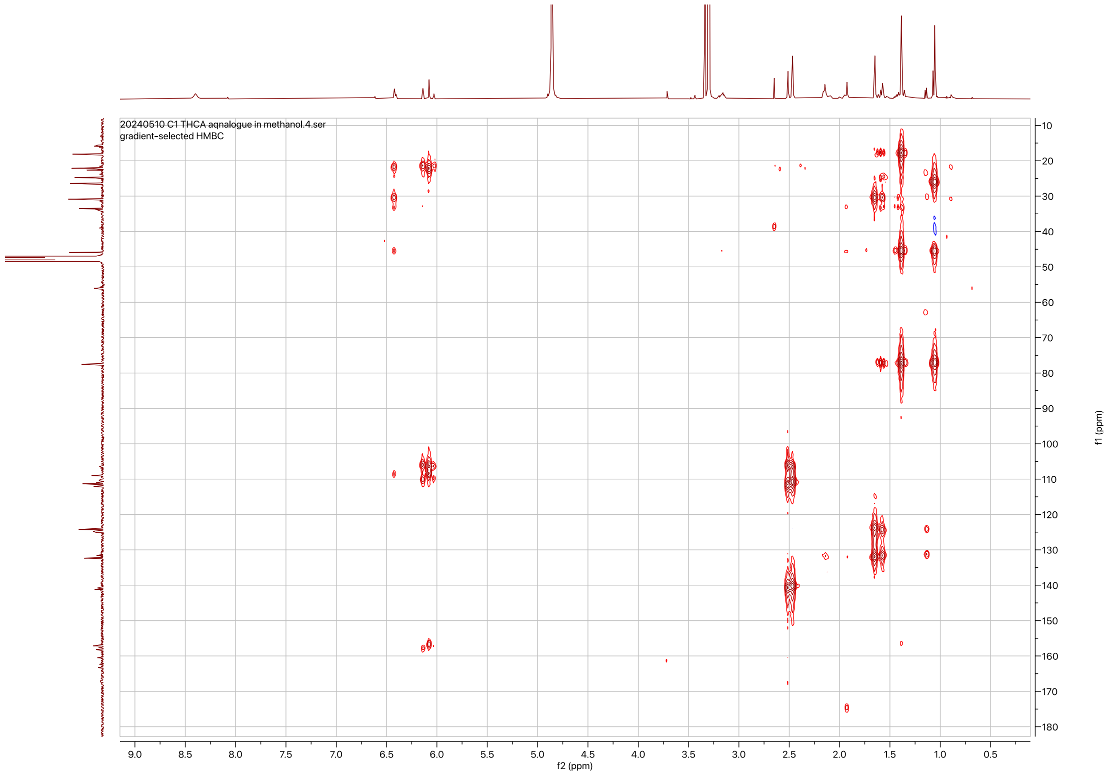

Figure S40 ^{1}H-^{13}C HMBC spectrum of compound Δ^{9}–THCCA in CD_3OD (500 MHz)

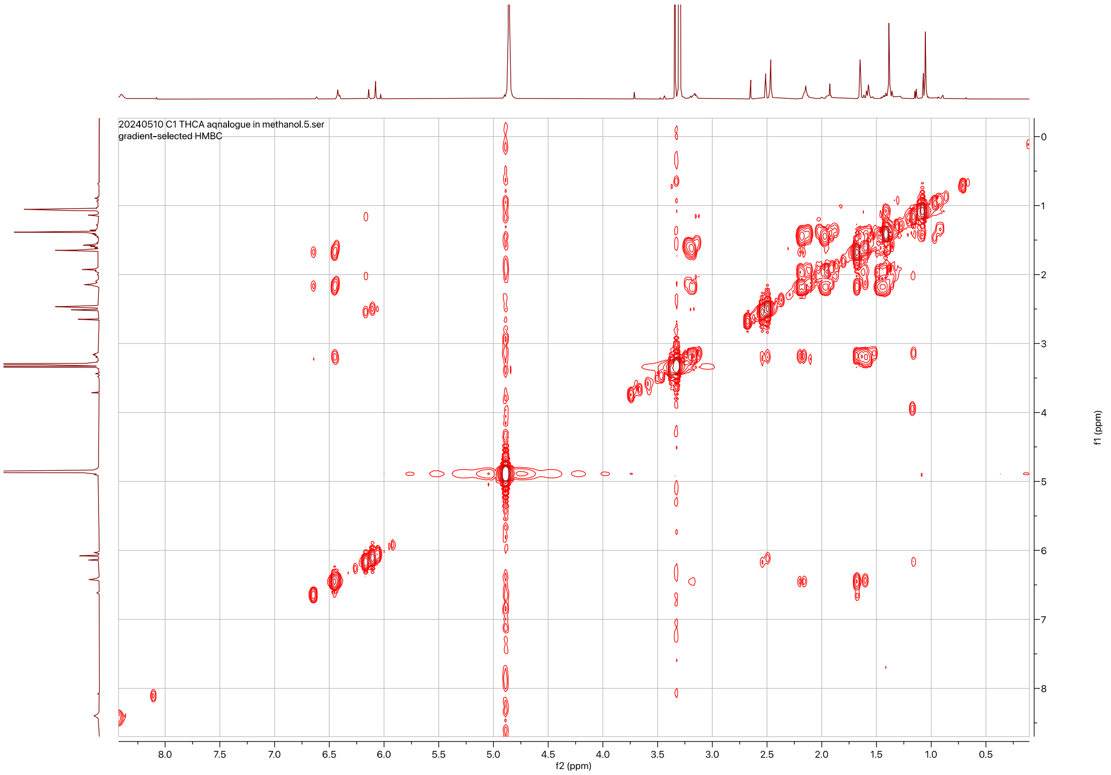

Figure S41 ^{1}H-^{1}H COSY spectrum of compound Δ^{9}–THCCA in CD_3OD (500 MHz)

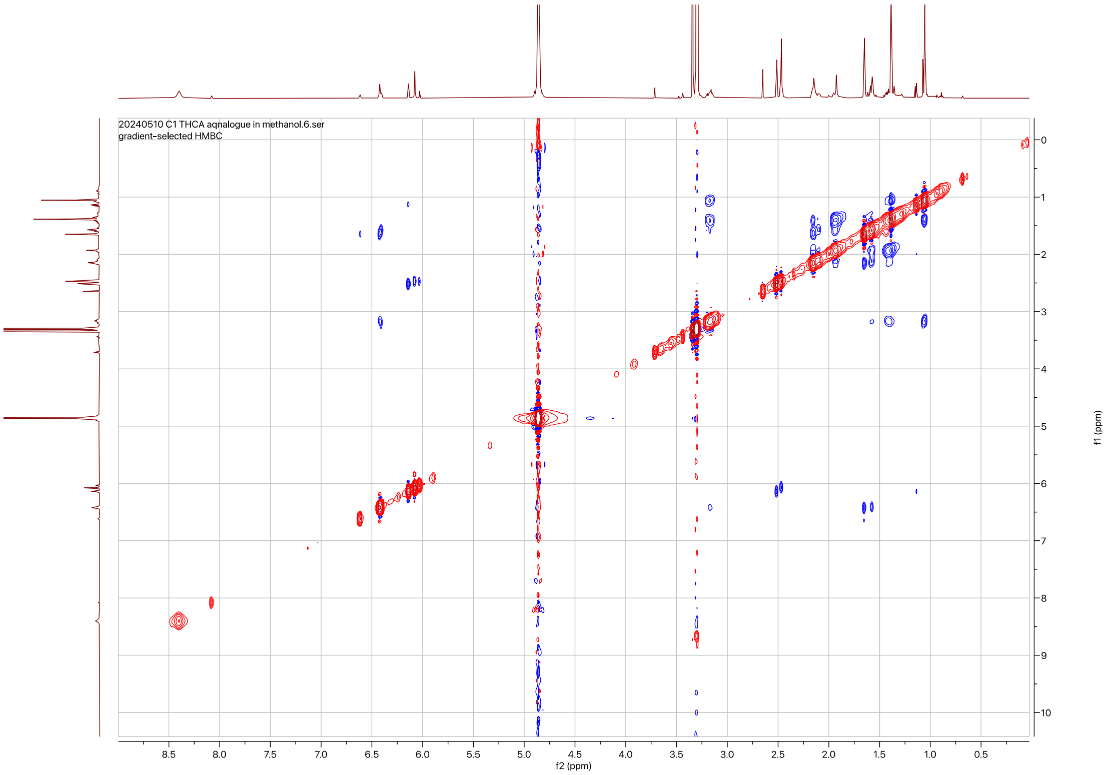

Figure S42 ^{1}H-^{1}H NOESY spectrum of compound Δ^{9}–THCCA in CD_3OD (500 MHz)

References

1. Yee, D. A.; Tang, Y. Investigating Fungal Biosynthetic Pathways Using Heterologous Gene Expression: Aspergillus Nidulans as a Heterologous Host. In *Methods in Molecular Biology*; Springer US: New York, NY, 2022; pp 41–52.
2. Harvey, C. J. B.; Tang, M.; Schlecht, U.; Horecka, J.; Fischer, C. R.; Lin, H.-C.; Li, J.; Naughton, B.; Cherry, J.; Miranda, M.; Li, Y. F.; Chu, A. M.; Hennessy, J. R.; Vandova, G. A.; Inglis, D.; Aiyar, R. S.; Steinmetz, L. M.; Davis, R. W.; Medema, M. H.; Sattely, E.; Khosla, C.; St. Onge, R. P.; Tang, Y.; Hillenmeyer, M. E. HEx: A Heterologous Expression Platform for the Discovery of Fungal Natural Products. *Sci. Adv.* **2018**, *4* (4).
3. Yee, D. A.; DeNicola, A. B.; Billingsley, J. M.; Creso, J. G.; Subrahmanyam, V.; Tang, Y. Engineered Mitochondrial Production of Monoterpenes in *Saccharomyces Cerevisiae*. *Metab. Eng.* **2019**, *55*, 76–84.
4. Jones, S.; Vignais, M.-L.; Broach, J. R. The *CDC25* Protein of *Saccharomyces Cerevisiae* Promotes Exchange of Guanine Nucleotides Bound to Ras. *Mol. Cell. Biol.* **1991**, *11* (5), 2641–2646.
5. Tang, S.; Wu, M. K. Y.; Zhang, R.; Hunter, N. Pervasive and Essential Roles of the Top3-Rmi1 Decatenase Orchestrate Recombination and Facilitate Chromosome Segregation in Meiosis. *Mol. Cell* **2015**, *57* (4), 607–621.
6. Cheng, W.; Li, W. Structural Insights into Ubiquinone Biosynthesis in Membranes. *Science* **2014**, *343* (6173), 878–881.
7. Yu, J.; Zhou, Y.; Tanaka, I.; Yao, M. Roll: A New Algorithm for the Detection of Protein Pockets and Cavities with a Rolling Probe Sphere. *Bioinformatics* **2010**, *26* (1), 46–52.
